# Interacting particle models on the impact of spatially heterogeneous human behavioral factors on dynamics of infectious diseases

**DOI:** 10.1371/journal.pcbi.1012345

**Published:** 2024-08-08

**Authors:** Yunfeng Xiong, Chuntian Wang, Yuan Zhang

**Affiliations:** 1 School of Mathematical Sciences, Beijing Normal University, Beijing, China; 2 Department of Mathematics, The University of Alabama, Tuscaloosa, Alabama, United States of America; 3 Center for Applied Statistics and School of Statistics, Renmin University of China, Bejing, China; Stockholms Universitet, SWEDEN

## Abstract

Human behaviors have non-negligible impacts on spread of contagious disease. For instance, large-scale gathering and high mobility of population could lead to accelerated disease transmission, while public behavioral changes in response to pandemics may effectively reduce contacts and suppress the peak of the outbreak. In order to understand how spatial characteristics like population mobility and clustering interplay with epidemic outbreaks, we formulate a stochastic-statistical environment-epidemic dynamic system (SEEDS) via an agent-based biased random walk model on a two-dimensional lattice. The “popularity” and “awareness” variables are taken into consideration to capture human natural and preventive behavioral factors, which are assumed to guide and bias agent movement in a combined way. It is found that the presence of the spatial heterogeneity, like social influence locality and spatial clustering induced by self-aggregation, potentially suppresses the contacts between agents and consequently flats the epidemic curve. Surprisedly, disease responses might not necessarily reduce the susceptibility of informed individuals and even aggravate disease outbreak if each individual responds independently upon their awareness. The disease control is achieved effectively only if there are coordinated public-health interventions and public compliance to these measures. Therefore, our model may be useful for quantitative evaluations of a variety of public-health policies.

## Author summary

This article investigates at multi-level the roles played by spatial heterogeneities in epidemic dynamics. For this purpose we propose a novel stochastic-statistical particle model implemented on a two-dimensional lattice field, where various individual-varying spatially inhomogeneous human behavioral factors are incorporated into an agent-based epidemic modeling framework.

With extensive simulations of our model, we are able to carry out thorough investigations on the impacts of heterogeneities at individual and spatial levels on epidemics trajectories during the early stage of an outbreak. It has been observed that spatial heterogeneity is consistently shown to be connected with suppressed and delayed epidemic peaks. These findings add valuable knowledge on the mechanism of the form and distribution of population clusters affecting an epidemic wave.

Moreover, we examine the interplay between spatial/individual heterogeneities and public-health policies in an intensive and systematic way across various parameter regimes, whose conclusions we believe are going to complement beneficially to both the theoretical and practical understanding of epidemic control.

## 1 Introduction

The COVID-19 pandemic, dubbed as ‘once-in-a-century health crisis’ [1], has made great impacts to the world. According to WHO Coronavirus (COVID-19) Dashboard, up to September 2023, there have been more than seven hundred million confirmed cases worldwide, including nearly seven million deaths reported. At the same time, people around the world have also learned important lessons from the pandemic which may enable us to be better prepared for future epidemic outbreaks. Among them, one of the key observations is that during the first episode of a disease outbreak, and in the absence of a remedy, early implementations of public-health interventions, such as social distancing, traveling restriction and public awareness programs, self-isolation, and quarantine, have been shown to be capable of delaying contagions and thus relieving the medical overrun [2–10]. What’s more, effects of these measures to alter progressions of pandemic spread depend heavily on the level of public compliances [11]. To better cope with newly emerging infectious disease pandemics that may bear characteristics similar to COVID-19, it is indispensable to make a clear understanding of interplays between outbreaks of transmissible diseases and natural and preventive human behavior [12]. To this end, modeling of human-disease interactions in a quantitative and predictive manner is urgently called for.

Despite rapid development in this area, there are still a number of open questions and unanswered challenges concerned with this topic. One of them is how to incorporate the spatial heterogeneity such as population contact structure and spatially heterogeneous interventions. It is well known that risks of disease spread may be augmented by natural behaviors such as gathering, commuting to work [13], and contacts with family, friends, co-workers, etc. (see e.g., [14]). However, assuming spatially homogeneous agent movement alone seems inadequate to explain aggregative characteristics of epidemic incidence, like spatial clustering [15, 16].

Another relevant topic is how to incorporate the public awareness of risks and uncertainties of an infectious disease, which may motivate individuals to change behaviors, e.g., avoiding places with observable signs of disease outbreaks [12, 17]. As a result, the chances of contacts between infectious and non-infectious individuals can be potentially reduced. Meanwhile, a large population migrating from places with a high level of prevalence, possibly driven by pandemic phobia, may propagate the disease outbreak. All of these behavioral changes described above play a key role in mitigating or escalating pandemic outbreaks [18]. Quantitative studies of preventive public reactions will enable us to assess effectiveness of various intervention options such as social distancing policy [2–4], and other public-health control measures [19].

To address the above issues, abundant real-life case studies of epidemic progression, including [20–26] with an intensive application of first-hand data, have been performed, with a common conclusion that environmental and socio-economic factors are capable to account for a major portion of the spatial variation in disease risk. For instance, the analysis of epidemic profiles in [20] attributes the spatial dependence to a combination of endogenous and exogenous processes. The study in [21] uncovers disparate outbreak processes in the spatio-temporal epidemic dynamics and the associated environmental factors, which exhibit a significantly high level of spatial heterogeneity displayed by commune attack rates. Moreover, geographical data processed with geographic information system (GIS) and spatial statistical tools have posited the importance of an interdisciplinary approach, with proactive measures, international solidarity and collaboration and a global perspective, to face with COVID-19 [24]. In particular, the georeferenced approach in [25] discovers that the synchronization of the local epidemic profiles is enhanced by addition of the dynamics of local commuting. [26] highlights the necessity of formulating regional measures and strategies for disease control and prevention in China, according to a geographically weighted regression model, which reflects the impacts of social and environmental factors that differ in region.

Numerous works have also aimed at establishing the connection between host-pathogen dynamics and environmental heterogeneity of fragmented landscapes [27, 28]. A major discovery is that the spatial pattern of transmission is the consequence of the subtle interactions between various types of social and physical heterogeneities such as exogenous and endogenous contagious processes, even though it appears to be a mere reflection of the dispersal and contact patterns among extant individuals. It is argued in [29] that social heterogeneity drives epidemics, namely, the predictive power and sophistication of the transmission-dynamics models will be greatly increased by the identification and measurement of heterogeneity of the social context, including behavior, mobility, structure, density, etc. The study in [30] clearly indicates the epidemiology has a non-negligible sensitivity of the mobility patterns of host individuals, and consequently stresses the importance of gathering information on such patterns. The metapopulation epidemic model integrated with agents’ memory of their traveling origin, proposed in [31], investigates the effects of self-imposed behavioral changes of individual mobility, which confirms the role of individual response to epidemic outbreaks in shaping epidemic spreading patterns. What’s more, in [28], the potential role of resource hotspots and resource provisioning is highlighted by a number of results of a spatially explicit SIR model integrated with movement ecology approaches, and the spatial organization of resources turns out to lead to nonlinear effects on infectious disease dynamics through alteration of host movement patterns as well as subsequent pathogen transmissions.

Now narrowing our attention to quantitative mathematical modeling and simulation studies, we find that there are mainly two classes of frameworks. Firstly, a vast number of works focuses on incorporating human mobility over a network from the (meta)-population level [25, 32–41]. Behavioral changes are incorporated by embedding an environment variable of awareness in a social network [12], coupling it with unaware-aware cycle which spreads awareness among nodes [10, 16, 42], or adding an “Alerted” compartment to account for a sub-population adopting preventive behaviors [41]. For these models, it is often assumed that the total population of the whole system or within each node in the meta-population model is fully mixed with all the agents acting identically. But this assumption may deviate from reality as a fully-mixed ODE model may yield a steeper and earlier first wave [7, 43]. The second class of modeling frameworks includes continuum mean-field PDEs from the macroscopic level and the kinetic modeling of the crowd dynamics on the mesoscopic scale (see e.g., [44–53]). These models account for both local transportation networks and the heterogeneity of population, and properly reflect the multi-scale features of epidemic dynamics [52]. Nonetheless, these continuum PDE models somehow treat the total population as a continuous field which diffuses like fluids, and assume each individual is able to contact with all others. This might not reflect the locality of individuals in a finite population, as each person can only contact a very limited number of agents from their social network. To the best of our knowledge, accurate modeling of spatial impacts on epidemic progressions still remains a significant challenge.

In this study, we endeavor to address the above challenges by formulating a mechanistic modeling and simulation studies of spatio-temporal geometry of human-disease interaction incidences, termed a stochastic-statistical environment-epidemic dynamical system (SEEDS). Specifically, an agent-based epidemic model at the individual microscopic level is proposed, coupled with evolutions of two environmental variables “popularity’’ and “awareness’’ on a 2-D lattice. Agents are assumed to take a random walk biased by natural and social behavior factors, such as aggregation behavioral patterns and public awareness of disease-transmission information. This will allow us to show that the frequent movement of individuals under high mobility rate, that reflects the strong interaction and correlation within them, indeed escalates the epidemic spread. By contrast, reducing the spread of location popularity can evidently contribute to formation of gathering events (spatial clustering), and impede the chances of contacting. As a direct consequence, a much flatter epidemic curve with smaller size of the first disease wave is observed. These provide a sound explanation of the role of spatial heterogeneity in epidemic dynamics, as the contact rate on the individual level and spatial transmission are highly influenced by the social influence locality [54].

More surprisingly, it is found that disease responses by more informed individual acting might not necessarily reduce their susceptibility. In fact, an increasingly vigilant to signs of an ongoing outbreak, like a trend of people traveling away from high-risk area, may even lead to aggravating disease outbreaks. Fortunately, coordinated disease interventions effectively control the outbreak. This coincides with our daily experience that disease control is achieved only if the whole community takes response in a collaborative way, instead of allowing each individual to respond independently upon their awareness. Therefore, our findings may pave the way for a more comprehensive understanding of effects of intervention options [55].

## 2 Models and methods

We will explore impacts of various human natural and preventive behaviors by three steps, and carry out investigations to compare effects of these spatially heterogeneous factors upon disease transmissions.

### 2.1 General setting of SEEDS

**Fig 1:**
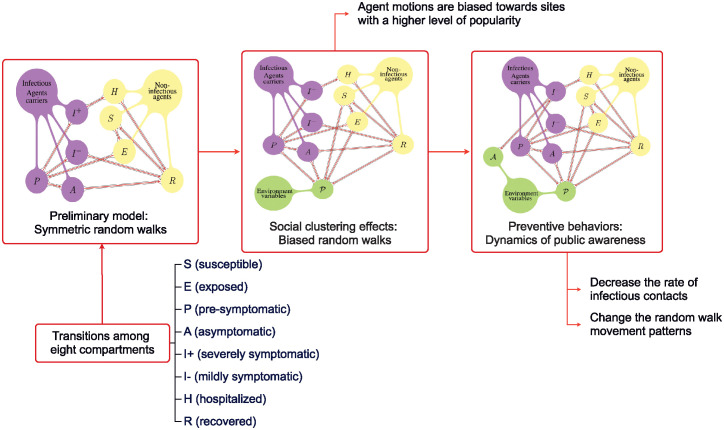
The mind map of our modeling. Preliminary model with symmetric movement (left), biased random walk foraging model with social clustering effects (middle) and the preventive behavior model with dynamics of public awareness (right).

The general assumption for our SEEDS builds on agent random walks in a dynamically evolving random environment set on a 2-D spatial lattice (graph). At the first step, a preliminary model as shown in the first panel of Fig 1 will be constructed assuming that agents take symmetric random walks over a 2-D lattice, while progressing among different states according to the transmissions and course of disease. For instance, infectious contacts (between susceptible and infected agents) could lead to disease transmissions and thus transitions between different states.

The second step follows by introducing a natural human behavioral factor through an environment variable defined on each lattice site (as shown in the second panel of Fig 1). Inspired by [56], where an environment variable defined on each lattice site quantifying attractiveness of the site, here in SEEDS we introduce a variable, denoted by P, to quantify the popularity of location. We assume that the popularity variable gets updated according to the “crowding effects”, which accounts for positive emotional responses associated with being in dense crowds [57–67]. Agent random walks are naturally assumed to be biased towards sites with a higher level of popularity.

The final step is to incorporate a preventive behavioral factor (as shown in the third panel of Fig 1). We introduce another environment variable, denoted by A, to reconstruct public awareness of disease transmission threats. It is updated according to local or global disease severity level. Particularly, prompted by alerts from public-health restriction policies, awareness should diffuse along with delivery of first-hand information about disease cases [12]. Meanwhile, along with release of alerts, or pandemic fatigue, where more and more people choose to lower risk perceptions [68–72], awareness may also naturally fades away.

We assume that A increases in accordance with severity of the epidemic outbreak. Furthermore, two different types of agent behavioral changes upon a raising of public awareness are investigated, that is, either the biasness or the rate of agent movement is assumed to be varied. We will refer to these two scenarios as Scenario I and Scenario II, respectively. Moreover, in Scenario II, two sub-cases are considered, assuming different ways in which the rate of agent movement is changed.

In Scenario I, disease-responses are assumed to be completely individualized. Naturally each agent chooses to actively avoid sites with visible signs of disease transmissions. Thus, agent random walks are directed and biased according to not only the local popularity level but also the local awareness level.

In Scenario II, instead of the biasness, we assume that the rate of random walks is affected by the awareness variable. Moreover, we also assume that awareness can be prompted by both symptomatic and non-symptomatic infectious agents. In fact, in many ways infectious populations without any visible symptoms can still provoke public awareness of disease transmissions; for example, real-time monitoring on SARS-CoV-2 can be achieved by analyzing wastewater data or public viral tests [73–75]. With the above assumptions, we study two sub-cases, where the first sub-case assumes that awareness level increases based on prevalence level of pre-symptomatic and asymptomatic infectious agents. Then in the second sub-case, public awareness is assumed to be uniform over the lattice field, rather than varying from site to site. Thus it can be modeled as a scalar function evolving over time. This assumption resembles the real-life situation when a coordinated and centralized epidemic monitoring system is available, leading to coordinated responses to disease transmissions.

In summary, an integrated dynamics of disease transmission events and human behavioral factors is obtained by human-environment-interaction modeling through a combined force of both environment variables that drives and biases agent random walks.

### 2.2 States and compartments

We assume that the total population is divided into the following states

$$\begin{array}{c} \left\{ \begin{array}{cc} & \text{S}\;\text{(susceptible)},\text{E}\;\text{(exposed)},\\ & \text{P}\;\text{(infectious}\;\text{and}\;\text{pre-symptomatic)},\text{A}\;\text{(infectious}\;\text{and}\;\text{asymptomatic)},\\ & \text{I}\;\text{(infectious}\;\text{and}\;\text{symptomatic)},\\ & \text{H}\;\text{(hospitalized)},\text{R}\;\text{(recovered}\;\text{and}\;\text{immune)}, \end{array}\right. \end{array}$$

where state I is further divided into two compartments depending on the symptom:

$$\begin{array}{c} \text{State}\;\text{I}\{\begin{array}{cc} & {\text{I}}^{\left(-\right)}\;\text{(mildly}\;\text{infectious}\;\text{symptomatic)},\\ & {\text{I}}^{\left(+\right)}\;\text{(severely}\;\text{infectious}\;\text{symptomatic}\;\text{and}\;\text{need}\;\text{hospitalization)}. \end{array} \end{array}$$

Here we assume that the E state is non-infectious, as in the real-world virus shedding does not necessarily begin right away after an exposure to the virus (see e.g. [8] for clinical evidence). Thus a new state P is introduced to resemble the stage from the beginning of virus shedding to onset of symptoms. Moreover, we assume that agents of states E, P, A, I^(−)^ and I^(+)^ are infectious active-virus carriers. Agents of states E, S, H, and R are assumed to be non-infectious. Furthermore, all the agents, except for the hospitalized ones, are mobile.

**Remark 1** *With a little abuse of notations*, S, E, P, A, I^(−)^ and I^(+)^, H, and R will be adopted to represent not only the state of an individual agent, but also the total number of agents belonging to that state on each site or even over the whole domain, that is, the corresponding compartment size.

**Remark 2** *Here we choose to exclude vital dynamics, as our focus is on transmission stages of a pandemic, and the death rate of COVID-19 is fairly low compared with the total population albeit catastrophic in absolute number of casualties (see relevant data in e.g*. [76]).

### 2.3 Spatially homogeneous unbiased random walk

We assume that the domain is rescaled to a unit square [0, 1] × [0, 1] with periodic boundary conditions, and all the mobile agents take random walks over a lattice grid defined on the domain. Without loss of generality, the deterministic total population size which is fixed over time is set to be *L*^2^ for some integer *L*; by choosing the spatial scaling factor as 1/*L*, the population density per unit area is exactly one. Denoting 1/*L* as *ℓ*, then the total population size is *ℓ*^−2^ and will be denoted as *N*_*ℓ*_, that is, *N*_*ℓ*_ = *ℓ*^−2^.

#### 2.3.1 Agent distribution over the 2D lattice

The numbers of agents of each of the eight states over the whole domain are denoted as *S*^*ℓ*^(*t*), *E*^*ℓ*^(*t*), *P*^*ℓ*^(*t*), *A*^*ℓ*^(*t*), *I*^(−), *ℓ*^(*t*), *I*^(+), *ℓ*^(*t*), *R*^*ℓ*^(*t*), and *H*^*ℓ*^(*t*), respectively, and the fractions of these quantities divided by *N*_*ℓ*_ are denoted as ${\bar{S}}^{\ell }(t)$, ${\bar{E}}^{\ell }(t)$, ${\bar{P}}^{\ell }(t)$, ${\bar{A}}^{\ell }(t)$, ${\bar{I}}^{(-),\ell }(t)$, ${\bar{I}}^{(+),\ell }(t)$, ${\bar{H}}^{\ell }(t)$, and ${\bar{R}}^{\ell }(t)$, respectively. We note that as population density per unit area is set as one, ${\bar{S}}^{\ell }(t)$ is not only the portion of compartment S within the whole population, but also density of the agents in S compartment per unit area. The same also applies to ${\bar{E}}^{\ell }(t)$, ${\bar{P}}^{\ell }(t)$, ${\bar{A}}^{\ell }(t)$, ${\bar{I}}^{(-),\ell }(t)$, ${\bar{I}}^{(+),\ell }(t)$, ${\bar{H}}^{\ell }(t)$, and ${\bar{R}}^{\ell }(t)$. The grid points of the lattice are denoted as **s** = (*s*_1_, *s*_2_), *s*_1_, *s*_2_ = *ℓ*, 2*ℓ*, ⋅⋅⋅, 1. As each agent is assigned to exactly one of the eight states mentioned above, every site **s** is attached with a vector recording the population of each compartment as follows:

$$\begin{array}{c} (S_{s}^{\ell }\left(t\right),E_{s}^{\ell }\left(t\right),P_{s}^{\ell }\left(t\right),A_{s}^{\ell }\left(t\right),I_{s}^{(-),\ell }\left(t\right),I_{s}^{(+),\ell }\left(t\right),H_{s}^{\ell }\left(t\right),R_{s}^{\ell }\left(t\right)). \end{array}$$ (1)

Moreover, we denote the number of mobile agents at site **s** at time *t* as $M_{\text{s}}^{\ell }(t)$, i.e.

$$\begin{array}{c} M_{s}^{\ell }\left(t\right):=S_{s}^{\ell }\left(t\right)+E_{s}^{\ell }\left(t\right)+P_{s}^{\ell }\left(t\right)+A_{s}^{\ell }\left(t\right)+I_{s}^{(-),\ell }\left(t\right)+I_{s}^{(+),\ell }\left(t\right)+R_{s}^{\ell }\left(t\right). \end{array}$$ (2)

#### 2.3.2 Individualized Poisson clocks and events which they govern

Occurrences of every individual event in such an agent-based model are kept tracked. Specifically, here we employ Poisson clocks to govern arrivals of events. These clocks set waiting times as independently exponentially distributed random variables whose expectations are the inverse of their advancing rates. Nine types of Poisson clocks (Type (I)—Type (IX)) are going to be employed, all of which are assumed to advance independently.

Hereafter *sc* is a temporal scaling parameter to help us explore how sociological factors interplay with the epidemiological ones. Subsequently, all the parameters assumed will be absolute constants independent of *sc* and *ℓ*. We note that below in the text, to better match real-world scenarios, *sc* is always chosen as 3.5 (Section 3.1.1). A sensitivity analysis is carried out in S1 Text to validate robustness with different values of *sc*.

**Symmetric random walks**. Type (I) clocks are set to govern agent movement. They advance according to independent Poisson processes with rate sc×D. A Type (I) Poisson clock is assigned to each mobile agent (that is, all the agents except for the H agents), and upon an advancement the corresponding agent will immediately jump from the current site, say **s**, to one of the nearest four neighboring sites with equal probability. In other words, for each mobile agent, the expected number of sites he visits per unit time is sc×D.

**Evolutions of agent states**. We assume six stages of evolutions of agent states according to the natural order of the course of disease, that is, infectious contacts, end of latent period, symptom onset, hospitalization, recovery, and immunity waning.

*Infectious contacts:* There are two types of disease transmission pathways: direct person-to-person infectious contacts, and indirect environment-to-human disease transmissions. The latter is also called the indirect pathway, that is, transmission from the environment which occurs via e.g. insects or by touching contaminated surfaces, etc. According to [77], for COVID-19 the risk of surface transmission was estimated to be 1,000 times lower than airborne transmission during a major outbreak. As a result, we hereby only take direct disease transmission pathways into consideration, in which case a susceptible individual can be infected only by another infectious individual residing in the same site.

A Type (II) Poisson clock with advancing rate λ is assigned to each pair of an S agent, and an infectious virus-active agent (i.e. an P, A, I^(−)^, or I^(+)^ agent), as long as these two are on the same lattice site. Upon a clock advancement, a contact between the two agents occurs, and the susceptible agent will progress to state E with probability 1 if the infectious agent is I^(−)^ or I^(+)^, and *β* if the infectious agent is P or A, where *β* is the reductive factor on the infectivity of asymptomatic carriers.

**Remark 3** *There is an equivalent description of the events governed by Type (II) clocks as mentioned above. On each infectious virus-active agent at site **s**, there is a master Poisson clock whose rate is proportional to the number of local mobile agents, i.e*., $\lambda M_{s}^{\ell }(t)$. *Upon an advancement of a master clock, the corresponding carrier will choose one randomly among all the mobile agents at the current site to contact; then disease transmission occurs (with the same probabilities mentioned above) whenever the agent whom the clock-carrier contacts happens to be a susceptible (S) agent. In summary, here in our model the rate of infections contacts at a certain site is proportional to the local population density, and sites with higher number of mobile agents are assigned with a higher rate of potential disease transmission. This leads to another distinct difference between our model and classical compartment models. The latter assume a fixed contact rate as a result of a homogeneously mixed population*.

*End of latent period:* Transitions at this stage are all governed by Type (III) Poisson clocks with rate *η*, one of which is assigned to each E agent, and advances at the end of the latent period. Upon an advancement, the corresponding E agent will progress to state P.

*Symptom onset:* Transitions at this stage are all governed by Type (IV) Poisson clocks with rate *η*′, the speed of symptom onset. Each P agent is assigned such a clock, which advances at the end of the pre-symptomatic period. Upon an advancement, the corresponding agent P will progress to state A with probability 1 − *ρ*. Otherwise, the P agent will become I^(−)^ (with probability *ρ*(1 − *p*_*H*_), or I^(+)^ (with probability *ρp*_*H*_), where *p*_*H*_ implies the probability of necessity of hospital treatment.

*Hospitalization:* We assume that only the I^(+)^ agents may possibly get hospitalized. On each I^(+)^ agent there is a Type (V) Poisson clock with rate $\delta_{I}^{\left(+\right)}$. Upon an advancement, the clock carrier transfers from state I^(+)^ to H.

*Recovery:* Transitions at this step can be divided into three types of events: direct recovery of asymptomatic infectious agents and of mildly symptomatic agents without hospitalizations and recovery of hospitalized agents.

A Type (VI), (VII), and (VIII) Poisson clock with rate *δ*_*A*_, $\delta_{I}^{\left(-\right)}$, and *δ*_*H*_ is assigned to each A agent, I^(−)^ agent, and H agent, respectively. Upon an advancement, the corresponding agent will progress to be an R agent.

*Immunity waning:* Transitions at this stage are all governed by Type (IX) Poisson clocks with rate *δ*_*R*_, which is assigned to each R agent. Upon an advancement, the corresponding agent progresses to be a susceptible agent.

All different types of events and their occurrence rates in the preliminary modeling setup are summarized in Table 1 (the superscript *ℓ* and the variable *t* are dropped for simplicity).

**Table 1:** Event types, state transitions, and corresponding transition rates of the preliminary agent-based symmetric random walk model.

Event types	State transitions	Rates
Random walks	−	sc×D
Infectious contacts	*S*_***s***_ + *P*_***s***_ → *E*_***s***_ + *P*_***s***_	*λβ*
	*S*_***s***_ + *A*_***s***_ → *E*_***s***_ + *A*_***s***_	*λβ*
	$S_{s}+I_{s}^{\left(-\right)}\to E_{s}+I_{s}^{\left(-\right)}$	λ
	$S_{s}+I_{s}^{\left(+\right)}\to E_{s}+I_{s}^{\left(+\right)}$	λ
End of latent period	*E*_***s***_ → *P*_***s***_	*η*
Symptom onset	*P*_***s***_ → *A*_***s***_	*η*′(1 − *ρ*)
	$P_{s}\to I_{s}^{\left(-\right)}$	*η*′*ρ*(1 − *p*_*H*_)
	$P_{s}\to I_{s}^{\left(+\right)}$	*η*′*ρp*_*H*_
Hospitalization	$I_{s}^{\left(+\right)}\to H_{s}$	$\delta_{I}^{\left(+\right)}$
Recovery	*A*_***s***_ → *R*_***s***_	*δ*_*A*_
	$I_{s}^{\left(-\right)}\to R_{s}$	$\delta_{I}^{\left(-\right)}$
	*H*_***s***_ → *R*_***s***_	*δ*_*H*_
Immunity waning	*R*_***s***_ → *S*_***s***_	*δ*_*R*_

#### 2.3.3 An analogous continuum ODE model

Continuum ODE system derived from the agent-based model with symmetric random walks described above can be used as an analogous continuum model. We denote $\bar{S}(t)$, $\bar{E}(t)$, $\bar{A}(t)$, ${\bar{I}}^{-}(t)$, ${\bar{I}}^{+}(t)$, $\bar{R}(t)$, and $\bar{H}(t)$ as the continuum versions of ${\bar{S}}^{\ell }(t)$, ${\bar{E}}^{\ell }(t)$, ${\bar{A}}^{\ell }(t)$, ${\bar{I}}^{(-),\ell }(t)$, ${\bar{I}}^{(+),\ell }(t)$, ${\bar{H}}^{\ell }(t)$, and ${\bar{R}}^{\ell }(t)$, respectively. Then by conservation of mass we obtain the following continuum ODEs where for simplicity the variable *t* is dropped:

$$\begin{array}{c} \left\{ \begin{array}{cc} & \frac{d\bar{S}}{dt}=-\lambda \left(\beta \left(\bar{P}+\bar{A}\right)+{\bar{I}}^{\left(-\right)}+{\bar{I}}^{\left(+\right)}\right)\bar{S}+\delta_{R}\bar{R},\\ & \frac{d\bar{E}}{dt}=\lambda \left(\beta \left(\bar{P}+\bar{A}\right)+{\bar{I}}^{\left(-\right)}+{\bar{I}}^{\left(+\right)}\right)\bar{S}-\eta \bar{E},\\ & \frac{d\bar{P}}{dt}=\eta \bar{E}-\eta^{\prime }\bar{P},\\ & \frac{d\bar{A}}{dt}=\eta^{\prime }\left(1-\rho \right)\bar{P}-\delta_{A}\bar{A},\\ & \frac{d{\bar{I}}^{\left(-\right)}}{dt}=\eta^{\prime }\rho \left(1-p_{H}\right)\bar{P}-\delta_{I}^{\left(-\right)}{\bar{I}}^{\left(-\right)},\\ & \frac{d{\bar{I}}^{\left(+\right)}}{dt}=\eta^{\prime }\rho p_{H}\bar{P}-\delta_{I}^{\left(+\right)}{\bar{I}}^{\left(+\right)},\\ & \frac{d\bar{H}}{dt}=\delta_{I}^{\left(+\right)}{\bar{I}}^{\left(+\right)}-\delta_{H}\bar{H},\\ & \frac{d\bar{R}}{dt}=\delta_{A}\bar{A}+\delta_{I}^{\left(-\right)}\bar{I^{\left(-\right)}}+\delta_{H}\bar{H}-\delta_{R}\bar{R}. \end{array}\right. \end{array}$$ (3)

### 2.4 Impacts of human natural behavior: Incorporating biased foraging behavior

In order to investigate the impacts of natural tendency of human behavior like foraging on epidemic progression, we incorporate an environment variable P representing popularity of that site perceived by nearby agents upon the agent-based symmetric random walk model described above (Section 2.3).

Specifically, P will be assigned to each site as a measurement of preference and willingness of mobile agents to visit the corresponding location. The value of P at each site **s** at time *t* is denoted as $P_{\text{s}}^{\ell }(t)$.

Moreover, we set in advance a static minimum value of P that can not be crossed over, denoted as $P_{\text{min}}$. This minimum value is used to resemble absolutely necessary activities such as getting food and other essential household goods, obtaining medical care, and traveling to perform essential work.

#### 2.4.1 Dynamics of the popularity variable

The popularity variable is assumed to get updated according to the crowding effects, a well-studied notion in behavioral sciences. That is, positive emotional responses associated with being in dense crowds arise widely in social activities ([61–67]). For instance, people tend to gain pleasure from places or events with high human density, like shopping malls and festivals, as the crowdedness of environment is an indicator of the presence of good reputation and service quality. In the meantime, Hotelling’s hypothesis [78] implies that clumping in a crowded area leads to increased popularity at adjacent places [78, 79], e.g., due to the additional job opportunities provided in this place. In other words, the popularity variable should be driven by its own dynamics, including spreading to neighboring sites, increasing along with clustering, and gradually losing its attractiveness.

**Increment and spread of popularity**. Crowded areas often enhances perceptions of food and service quality, or contribute positively to consumer’s quality inferences ([57–60]). Moreover, because of the agglomeration of similar retailer ([78–80]), once a store becomes over-crowded and out of capacity, people choosing to leave will very likely move to adjacent places for the same shopping purposes. As a result, the neighbourhood of a popular location often experience popularity increase. Therefore, it is reasonable to assume that P increases with rates in proportion to the number of local mobile agents who have no visible symptoms; at the same time, P spreads over nearby neighborhood.

On each mobile agent who has no visible symptoms, i.e., the group of S, E, P, A, and R agents, there is a Type (X) Poisson clock with rate $I_{P}\times \ell^{-1}\times sc$. Upon an advancement at time *t*^−^ and site **s**, $P_{\text{s}}(t)$ increases by a magnitude of ${\bar{\delta }}_{P}^{+}\ell$.

A Type (XI) clock with rate $\Lambda_{P}\times sc$ is assigned to every site and upon an advancement at *t*^−^ at site **s**, popularity of site **s** is updated according to the value at *t*^−^ and that of its four nearest neighbors with a magnitude of *α*, *α* ∈ (0, 1), i.e. $P_{\text{s}}^{\ell }(t^{-})$ is updated to be

$$\begin{array}{c} P_{s}^{\ell }\left(t\right)=P_{s}^{\ell }\left(t^{-}\right)+\frac{\alpha }{4}\ell^{2}\Delta^{\ell }P_{s}^{\ell }\left(t^{-}\right), \end{array}$$ (4)

where Δ^*ℓ*^ is the discrete spatial Laplacian operator associated with the lattice grid, namely,

$$\begin{array}{c} \Delta^{\ell }P_{s}^{\ell }\left(t\right)=\ell^{-2}(\sum_{s^{\prime }s^{\prime }∼s}P_{s^{\prime }}^{\ell }\left(t\right)-4P_{s}^{\ell }\left(t\right)), \end{array}$$ (5)

where **s**′ ∼ **s** indicates all of the neighboring sites of **s**.

**Fading of popularity**. For population attractions such as stores, restaurants, etc., popularity of the site generally decreases once variety seeking of customers is triggered by boredom induced by repeated purchases [81]. So we assume that P decreases at a constant rate independent of number of local agents.

Let ${\bar{\delta }}_{P}^{-}$ and $D_{P}$ be constants determining the speed of the decay. A Type (XII) clock with rate $D_{P}\times sc\times \ell^{-1}$ is assigned to every site and upon its advancement at site **s** and time *t*^−^, $P_{\text{s}}^{\ell }(t^{-})$ is immediately updated to be the larger value between $P_{\text{min}}$ and the following quantity:

$$\begin{array}{c} P_{s}^{\ell }\left(t^{-}\right)(1-\frac{{\bar{\delta }}_{P}^{-}}{\ell^{-1}}). \end{array}$$ (6)

#### 2.4.2 Biased random walks guided by popularity

We now change the assumptions of symmetric random walks (Section 2.3.2) into biased ones towards areas with higher values of P. More precisely, upon an advancement of a Type (I) clock at time *t*^−^, the corresponding agent will immediately jump from current site, say **s**, to one of the neighboring sites, say **k**, with a probability $q_{\text{s}\to \text{k}}^{\ell }(t)$ defined as follows:

$$\begin{array}{c} q_{s\to k}^{\ell }\left(t\right):=\frac{P_{k}^{\ell }\left(t^{-}\right)}{\sum_{s^{\prime }s^{\prime }∼s}P_{s^{\prime }}^{\ell }\left(t^{-}\right)}. \end{array}$$ (7)

### 2.5 Impacts of human preventive behavior: Incorporating public awareness of disease

Based on the biased random walk foraging-behavior model constructed above (Section 2.4), we will further explore impacts of public-awareness of disease on epidemic dynamics. In particular, a second environment variable A representing public awareness of threats of disease transmissions is introduced into the model. At the individual level, each agent may change their moving pattern according to their assessment of severity of prevalence. In contrast, risks and uncertainties of an infectious disease can also prompt public measures like travel restrictions, and area lock-down. To imitate the real-life experience, two scenarios, called Scenarios I and II, will be considered, together with two sub-cases in Scenario II, which will be called Scenario II-i and II-ii.

**(1)** Scenario I: We assume that A will affect the attractiveness of a site, without changing the moving rate of mobile agents. Moreover, A is assumed to receive an increase in the presence of local symptomatic infectious agents as well as hospitalization events. This scenario reconstructs the response of taking individualized actions to avoid places with visible high prevalence, in the absence of effective control of population mobility.
**(2)** Scenario II-i: Here it is the agent movement rates that are assumed to change in a local manner with A. Futhermore, awareness growth upon appearances of hospitalization events and local symptomatic as well as asymptomatic infectious agents is accounted for, which resembles an active disease monitoring system. This scenario aligns with public policies, such as travel restrictions, aimed at reducing population mobility based on individuals’ perceptions of disease transmission in their current locations.
**(3)** Scenario II-ii: Here A is assumed to evolve macroscopically according to the overall prevalence level of the whole community. This scalar value is assumed to uniformly affect the rate of agent movement, and grow upon appearances of both symptomatic and asymptomatic agents (at any site in the system). This scenario corresponds to coordinated response measures which are continuously updated according to the epidemic prevalence level in the whole community.

#### 2.5.1 Preliminary assumptions of the awareness variable

At each site **s** at time *t* the value of A is denoted as $A_{\text{s}}^{\ell }(t)$. We assume that $A_{\text{s}}^{\ell }(t)\in [0,1]$ for every **s** and *t*. Moreover, when $A_{\text{s}}^{\ell }(t)=0$, agents are completely unaware of the disease transmission at **s**, and their movement is guided by the local popularity variable alone. In contrast, when $A_{\text{s}}^{\ell }(t)=1$, agents are assumed to completely avoid traveling to site **s**.

#### 2.5.2 Scenario I: Integrating disease awareness into direction of agent movement patterns

In this scenario, we assume that directions of random walks depend on both the popularity and the awareness variables. Moreover, it is assumed that A receives an increase in the presence of symptomatic infectious agents and also at each hospitalization event.

**Biased random walk guided by awareness**. Based on the biased random walk foraging model, we make changes to the events governed by Type (I) clocks (Section 2.3.2). Upon an advancement of a Type (I) clock at time *t*^−^, the corresponding agent will immediately jump from current site, say **s**, to one of the neighboring sites, say **k**, with a probability $q_{\text{s}\to \text{k}}^{\ell }(t)$ defined as follows:

$$\begin{array}{c} q_{s\to k}^{\ell }\left(t\right):=\frac{\left(1-A_{k}^{\ell }\left(t^{-}\right)\right)P_{k}^{\ell }\left(t^{-}\right)}{\sum_{s^{\prime }s^{\prime }∼s}(\left(1-A_{s^{\prime }}^{\ell }\left(t^{-}\right)\right)P_{s^{\prime }}^{\ell }\left(t^{-}\right))}. \end{array}$$ (8)

In this manner, an agent now will actively avoid sites with high visible prevalence level, while their total moving rate remains unchanged.

**Dynamics of awareness**. A natural phenomenon in a prolonged public health crisis is pandemic fatigue due to various reasons such as implementation of invasive measures ([68–71]). Those who have developed pandemic fatigue will decrease their effort to follow recommendations and restrictions, and insufficiently keep themselves informed about prevalence, hospital capacity, etc. What’s more, information contained in first-hand observation and by word of mouth tend to suffer degradation in spread, and will lead to less determined reaction [12]. To this end, we assume that the awareness variable deceases and could also diffuse and decay spontaneously, while increasing upon occurrences of local hospitalization events, and also at a rate in proportion to the number of local patients with visible symptoms.

*Decay and spread of the awareness variable:* Let *θ*^−^ and $D_{A}$ be constants determining the speed of decay of A. A Type (XIII) clock with rate $D_{A}\times \ell^{-1}\times sc$ is assigned to every site, and upon its advancement at site **s** and time *t*^−^, $A_{\text{s}}^{\ell }(t^{-})$ immediately receives a decrease by a proportion of *ℓθ*^−^.

A Type (XIV) clock with rate $\Lambda_{A}\times sc$ is assigned to every site. Upon its advancement at **s** and time *t*, public awareness of the nearest four neighbouring sites spreads to **s** with a magnitude of *η*_3_ ∈ (0, 1), i.e. $A_{\text{s}}^{\ell }(t^{-})$ is updated to be

$$\begin{array}{cc} A_{s}^{\ell }\left(t\right) & =A_{s}^{\ell }\left(t^{-}\right)+\frac{\eta_{3}}{4}\ell^{2}\Delta^{\ell }A_{s}^{\ell }\left(t^{-}\right). \end{array}$$ (9)

*Increase of the awareness variable:* Whenever an agent of state *I*^(+)^ at site **s** and time *t* is hospitalized, $A_{\text{s}}^{\ell }(t^{-})$ immediately receives an increase as follows:

$$\begin{array}{c} A_{s}^{\ell }\left(t\right)=A_{s}^{\ell }\left(t^{-}\right)\left(1-\theta_{r}^{+}\right)+\theta_{r}^{+}, \end{array}$$ (10)

where $\theta_{r}^{+}\in (0,1)$ is a constant. Meanwhile, a (XV) clock Poisson clock with rate $I_{A}\times \ell^{-1}\times sc$ is attached to each symptomatic agent (including both *I*^(−)^ and *I*^(+)^ agent). Upon an advancement at site **s** and time *t*^−^,

$$\begin{array}{c} A_{s}^{\ell }\left(t\right)=A_{s}^{\ell }\left(t^{-}\right)\left(1-\ell \theta_{a}^{+}\right)+\ell \theta_{a}^{+}, \end{array}$$ (11)

where $\theta_{a}^{+}$ is a constant measuring the magnitude of such an increment.

**Remark 4** *We note that the dynamics described above guarantees that* $A_{s}^{\ell }(t)\leq 1$ *for every **s** and every t*.

#### 2.5.3 Scenario II-i: Suppressing moving rate by locally-supervised disease awareness

In Scenario II, instead of biasness we assume that it is the rate of random walks that will change according to A. Furthermore, in Scenario II-i, we incorporate public health measures based on a local monitoring system which can also detect the prevalence level of infectious virus carriers without significant symptoms.

We assume that the rate of agent movement reduces upon the local awareness level. More precisely, based on the biased random walk foraging model, we make changes to the rate of Type (I) clock (Section 2.3.2) to be site-dependent as follows:

$$\begin{array}{c} (1-A_{s}^{\ell }\left(t\right))D\times sc. \end{array}$$ (12)

Meanwhile, the biasness of random walks is assumed to follow the same assumption as the foraging biased random walk model, that is, the biasness depends only on P as in (8). In summary, an increase of disease transmission risk of local area leads to a decrease of agent mobility.

**Supervision-prompted awareness**. Moreover, the assumptions of dynamics of A is different from those in Scenario I (see Section 2.5.2). Namely, we assume that not only infectious symptomatic agents but also the presymptomatic and asymptomatic infectious agents will prompt awareness. This assumption resembles the situation if there is an epidemic monitoring system which can provide an estimation on the prevalence level through Polymerase chain reaction (PCR) sample survey or sewage monitoring.

Specifically, recall that back in Section 2.5.2 a (XV) clock is attached to each symptomatic agent (including both I^(−)^ and I^(+)^ agent). Now in order to complete the setup of Scenario II-i, we assume that not only each symptomatic agent, but also each P and each A agent is assigned a Type (XV) Poisson clock. Upon an advancement the local awareness variable increases in the same way as in Eq (11).

#### 2.5.4 Scenario II-ii: Suppressing moving rate by spatially-uniform awareness

In Scenario II-ii, we introduce a coordinated public health measure in this hypothetic disease control campaign where information on different sites are shared to derive a uniform awareness factor. In contrast to the scenarios described above which adopt spatially-varying awareness, we instead assume a scalar-valued awareness. Namely, for every **s**, $A_{\text{s}}^{\ell }(t)\equiv A^{\ell }(t).$ We still assume that $A^{\ell }(t)$ is between 0 and 1.

This scenario takes place when a coordinated monitoring system is available, such that a coordinated disease control campaign is implemented in this whole region and is updated dynamically according to the level of prevalence. Motivated by this real-world scenario, we assume that the rate of agent movement reduces upon increased value of $A^{\ell }(t)$. More specifically, changes are made to the rate of Type (I) clock (Section 2.3.2) as follows:

$$\begin{array}{c} (1-A^{\ell }\left(t\right))D\times sc. \end{array}$$ (13)

**Dynamics of awareness**. Awareness dynamics here shares similarity to that in Scenario II-i (Section 2.5.3).

Firstly, a merged Type (XIII) clock is set to govern the decrease of the scalar-valued awareness variable. The merged Type (XIII) clock advances with rate $D_{A}\ell^{-2}\times sc$. Upon an advancement at time *t*^−^, $A^{\ell }(t^{-})$ immediately receives a decrease by a proportion of *ℓ*^2^*θ*^−^.

Secondly, whenever an I^(+)^ agent at any site and time *t* is hospitalized as described in Section 2.3.2, $A^{\ell }(t^{-})$ receives an increase as follows:

$$\begin{array}{c} A^{\ell }\left(t\right)=A^{\ell }\left(t^{-}\right)\left(1-\ell^{2}\theta_{r}^{+}\right)+\ell^{2}\theta_{r}^{+}, \end{array}$$ (14)

where $\theta_{r}^{+}\in (0,1)$ is a constant as in (10).

Thirdly, we adopt a similar assumption to that of supervision-prompted awareness as in Scenario II-i (Section 2.5.3). More precisely, a merged Type (XV) clock is set to advance with rate

$$\begin{array}{c} sc\times (P^{\ell }\left(t\right)+A^{\ell }\left(t\right)+I^{\ell }\left(t\right)). \end{array}$$ (15)

Upon an advancement at time *t*^−^, the scalar-valued awareness variable will immediately receive an increase as follows:

$$\begin{array}{c} A^{\ell }\left(t\right)=A^{\ell }\left(t^{-}\right)\left(1-\ell^{2}\theta_{a}^{+}\right)+\ell^{2}\theta_{a}^{+}, \end{array}$$ (16)

where $\theta_{a}^{+}$ is a constant as in (11).

**Remark 5** *We note that in* (14), *it is necessary to keep the term ℓ*^2^ *in this equation. This is because this type of update is set to happen whenever a hospitalization event occurs, at every site in the system. Thus there are a total of order ℓ*^−2^ *competing Poisson clocks, each of which advances at an absolute rate independent of ℓ, which yields a merged total rate of a comparable order of magnitude to ℓ*^−2^.

## 3 Results and simulations

### 3.1 Simulations for agent-based symmetric random walk model and continuum ODE

In this section, we compare simulations of the agent-based unbiased random walk model (Section 2.3) and its analogous continuum ODE model (Section 2.3.3). Simulations indicate that with suppressed moving rate, spatial heterogeneity emerges and significantly impedes formation of disease wave peaks.

#### 3.1.1 Parameter values

For the agent-based simulations, we use parameter values that are set according to the features of COVID-19 pandemic, especially the omicron variant, which are referred to literatures such as [8, 82–87]. All the parameter values are displayed in Table 2 below.

**Table 2:** Event types, parameter values, sources and references, and corresponding physical meanings in the mathematical model for the preliminary agent-based symmetric random walk model.

Event types	Parameters values	Physical meanings	Sources and references
Random walks	*ℓ* = 1/100	spatial scaling factor	
	*sc* = 3.5	temporal scaling parameter	
	D =0.25, 0.5, 1, 2	agent moving rate	
Infectious contacts	*R*_0_ = 8.2	basic reproduction number	Ref. [87]
	λ = 1.018	rate of infection onset	Calibrated by Eq (17)
	*β* = 1	reductive factor on infectivity of asymptomatic carriers	Ref. [8]
End of latent period	*η* = 1/(1.2 days)	inverse of latent period length	Ref. [8]
Symptom onset	*η*′ = 1/(1.8 days)	rate of symptom onset	Refs. [8] [85]
	*ρ* = 0.745	probability of symptomatic infectious cases	Ref. [83]
Hospitalization	*p*_*H*_ = 0.0272	probability of hospitalization	Ref. [8]
	$\delta_{I}^{+}=1/(3.8\;\text{days})$	rate of hospitalization onset	Ref. [82]
Recovery	$\delta_{I}^{-}=1/(7.5\;\text{days})$	rate of virus removal	Ref. [86]
	*δ_A_* = 1/(7.5 days)	rate of virus removal	Ref. [86]
	*δ_H_* = 1/(6 days)	inverse of recovery period	Ref. [8]
Immunity waning	*δ_A_* = 1/(268 days)	inverse of immunity waning period	Ref. [84]

Particularly, for choice of λ, rate of infection after infectious contacts, we resort to a computation based on values of parameters that are easier to observe. The reason is that λ, a public-health parameter rather than physiological clinical parameter, depends not only on the transmission characters of the disease itself, but also various other factors, such as social norms, living habits, public transportations, etc. To this end, we choose to utilize the following estimation formula of the basic reproduction number *R*_0_ depending on λ, such that a suitable choice of *R*_0_ leads to the value of λ.

$$\begin{array}{c} R_{0}=\lambda [\underset{\text{asymptomatic}}{\underset{︸}{\beta \left(1-\rho \right)(\frac{1}{\eta^{\prime }}+\frac{1}{\delta_{A}})}}+\underset{\text{mildly}\;\text{symptomatic}}{\underset{︸}{\rho (\frac{1}{\eta^{\prime }}+\frac{1}{\delta_{I}^{-}})}}]. \end{array}$$ (17)

According to real world estimations of *R*_0_ of omicron variant (see e.g. [87]), here we choose *R*_0_ to be 8.2.

To select the value of *δ*_*R*_ (the inverse of the immunity protection waning period), we use estimation and extrapolation by stretched exponential fitting based on the data of protective immunity (95% confidence interval) of previous SARS-CoV-2 infection against medically attended symptomatic omicron XBB reinfection in Singapore (see Table 3 in [84]).

Furthermore, we estimate *p*_*H*_ by finding the weighted average of age-dependent proportion of un-vaccinated symptomatic infections requiring hospitalization the hospitalization rate among the symptomatic population stratified by age in China (see Supplementary Table 2 and Table 8 in [8]), where the weights are set according to the approximated age distribution of the total population of China. Particularly, we obtain the hospitalization rate over all infections by multiplying the age-dependent proportion of un-vaccinated infections who developed symptoms with age-dependent proportion of un-vaccinated symptomatic infections requiring hospitalizations (see Supplementary Table 4 and Table 8 in [8]).

#### 3.1.2 Initial data

The initial data of agent-based and continuum simulations are set as a small perturbation from the non-disease equilibrium with a relatively negligible fraction of active-virus carriers, i.e., the E, P, A, I^(−)^, and I^(+)^ agents. The initial data of H and R agents are set as zero (uniformly everywhere in the domain) in all simulations. Moreover, in agent-based simulations, we always keep the total population as *N*_*ℓ*_ at all times. Note that *ℓ* is hereby fixed as 1/100 while yields the value of *N*_*ℓ*_ to be 10000.

For S, E, P, A, I^(−)^, and I^(+)^ agents, we set up their initial values by first sampling a multi-valley Gaussian function (19) over every point **x** = (*x*_1_, *x*_2_) ∈ [0, 1] × [0, 1] and rounding to integers. Then we find their renormalization over the domain, and set these as the initial value for the simulations of the continuum ODE model.

Denote by the initial value of S, E, P, A, I^(−)^, and I^(+)^ agents over every point **x** ∈ [0, 1] × [0, 1] as *S*(**x**, 0), *E*(**x**, 0), *P*(**x**, 0), *A*(**x**, 0), *I*^(−)^(**x**, 0), and *I*^(+)^(**x**, 0), respectively. We set

$$\begin{array}{c} \begin{array}{cc} S(x,0) & =1-\delta_{1}\left(x\right)-\delta_{2}\left(x\right)-\delta_{3}\left(x\right)-\delta_{4}\left(x\right)-\delta_{5}\left(x\right),\\ E(x,0) & =\delta_{1}\left(x\right),P\left(x,0\right)=\delta_{2}\left(x\right),A\left(x,0\right)=\delta_{3}\left(x\right),\\ I^{\left(-\right)}\left(x,0\right) & =\delta_{4}\left(x\right),I^{\left(+\right)}\left(x,0\right)=\delta_{5}\left(x\right), \end{array} \end{array}$$ (18)

where the perturbation *δ*_*i*_(**x**) is composed of 30 independent Gaussian functions with randomly chosen centre positions $\left(x_{1}^{i},x_{2}^{i}\right)$, heights *h*_*i*_ and widths *σ*_*i*_ set as follows for *i* = 1, 2, 3, 4, 5:

$$\begin{array}{c} \begin{array}{cc} \delta_{i}\left(x\right) & \propto \sum_{i=1}^{30}\sum_{j=-L}^{L}\sum_{k=-L}^{L}\;h_{i}\;\text{exp}\;(\frac{-{\left(x_{1}-x_{1}^{i}+j\right)}^{2}-{\left(x_{2}-x_{2}^{i}+k\right)}^{2}}{\sigma_{i}}) \end{array} \end{array}$$ (19)

where $h_{i}=0.001r_{i}^{\left(1\right)},\sigma_{i}=0.01r_{i}^{\left(2\right)},(x_{i},y_{i})=(r_{i}^{\left(3\right)},r_{i}^{\left(4\right)})$ and $r_{i}^{\left(1\right)},r_{i}^{\left(2\right)},r_{i}^{\left(3\right)},r_{i}^{\left(4\right)}$ are samplings of independent uniform random number in [0, 1); the periodic neighborhood are added to ensure the periodic boundary condition of all agent and field variables (*L* = 20). For more details, see the Matlab codes available online.

**Remark 6** *It is worth noting that with the above setup, in agent-based simulations, population density per unit area is conserved as one as desired*.

#### 3.1.3 Daily cases and 2D portraits of agent distributions

**Fig 2:**
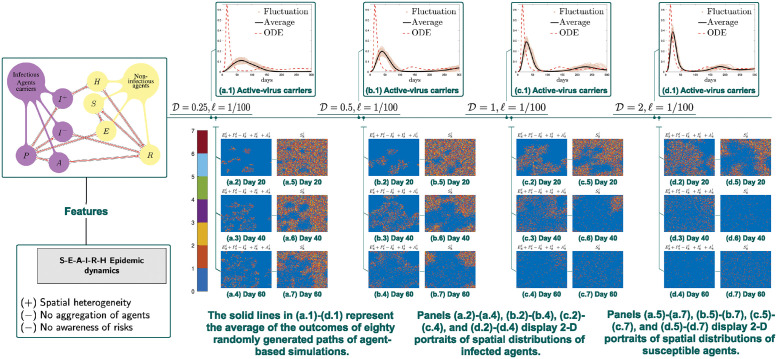
Simulations of symmetric random walk model. Panels (a.1)-(d.1) compare ${\bar{E}}^{\ell }(t)+{\bar{P}}^{\ell }(t)+{\bar{A}}^{\ell }(t)+{\bar{I}}^{(-),\ell }(t)+{\bar{I}}^{(+),\ell }(t)$. Increasing the rate D of agent movement from 0.25 to 0.5 to 1 to 2 leads to a heightened disease outbreak, and thus escalate severeness of the first episode of the epidemic.

**Fig 3:**
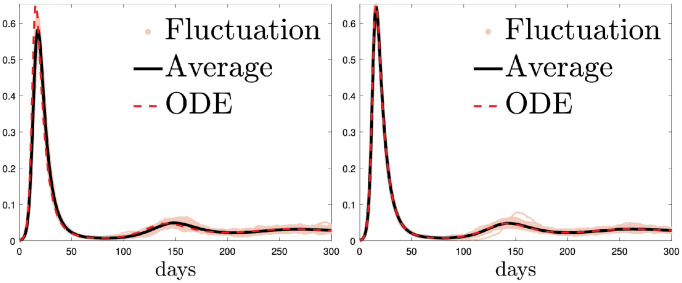
The continuum ODE is better approximated by the corresponding agent-based unbiased random walk model when the mobility speed D further increases. Parameters and initial data are the same as those used in Fig 2(a.1), 2(b.1), 2(c.1) and 2(d.1), except for that D is changed to 10 (left) or 100 (right). Note that *ℓ* is still fixed as 1/100. The solid black line represents the average of eighty randomly generated paths of the agent-based model, and the red dashed line represents the outcome of the continuum ODEs.

Compared with agent-based simulations, due to lack of spatial heterogeneity introduced by randomness, the corresponding continuum simulations tend to display a heightened disease outbreak (Fig 2(a.1)–2(d.1)) and thus escalated severeness of the first episode of the pandemic. What’s more, increasing the rate of agent movement also appears to have the same effects and bring the agent based model toward our ODE. Specifically, epidemic spread accelerates along with an increase of rate of agent movement (Fig 2(a.1)–2(d.1) and Fig 3). What we find in spatial-temporal clustering patterns of susceptible agents further supports our conjecture, that is, over-estimation of classical ODE compartment models of disease peaks stems from negligence of locality of agent movements rather than the finite size effects (see Fig 2(a.5)–2(a.7), 2(b.5)–2(b.7), 2(c.5)–2(c.7) and 2(d.5)–2(d.7)). Indeed with a fixed *ℓ* and *N*_*ℓ*_, when D is sufficiently large (see Fig 3 for the case when D=10 (left), and D=100 (right)) we obtain a good agreement between the agent-based and continuum ODE models. This indicates that finite size effects does not play an substantial role in creating the difference between compartment and agent based model. In reality, individual moving speed is always finite, and the contact range around an active-virus carrier is limited. Such social influence locality prevents those who are susceptible to be simultaneously infected. As a result, ODE models predicts a much higher disease transmission rate compared to that in reality.

At the same time, reducing the rate of agent movement can be interpreted as uniform and universal traffic restrictions. Thus our observations provide quantifications of effects of this type of public-health policies. Moreover, the simulation results with a dependence on agent walk speed account for the discrepancy in outbreaks at different historical times. Namely, epidemic spread has been significantly accelerated as transportation tools become more advanced, especially when long-distance and world-wide travels are increasingly more popular. As a sharp comparison, occurring in the preindustrial age, Black Death plague was boosted into a large-scale pandemic with a much slower speed compared to COVID-19.

In Fig 2(a.1)–2(d.1), as a comparison to agent-based simulations, the associated continuum simulations exhibit accelerated and heightened diseases peaks. These epidemic aggravation impacts also emerge as D increases, as displayed in Fig 2(a.5)–2(a.7), 2(b.5)–2(b.7), 2(c.5)–2(c.7) and 2(d.5)–2(d.7). The spreading of epidemic, i.e. the rate of susceptible agents transitioning to other states (E, P, A, I^(−)^, I^(+)^, H, or R), grows faster along with an increase of D. It is worth noting that the ‘cavity’, i.e., lower density regions of susceptible agents at a certain time point, stands for the spatial area affected by the epidemic at that time. Indeed, at day 20 (see Fig 2(a.5)–2(d.5)), larger cavity gradually appears as D increases. The phase diagram is displayed till day 60, which covers the time duration of the first disease wave. Additionally, for completeness of simulation output, two-dimensional portraits of spatial distributions of active-virus carriers are displayed in the remaining panels in Fig 2.

**Remark 7** *We note that at day 60, a significantly lower spatial density of* $P_{s}^{\ell }(t)+A_{s}^{\ell }(t)+I_{s}^{(-),\ell }(t)+I_{s}^{(+),\ell }(t)$ *emerges as* D increases to 2 (Fig 2(c.4) and 2(d.4)); *the reason that is that the first wave has already passed by day 60 in this case (see also (c.1) and (d.1)). This is a situation where the disease peak occurs considerably earlier than day 60*.

### 3.2 Simulations of biased foraging model: Effects of popularity-induced aggregation

**Fig 4:**
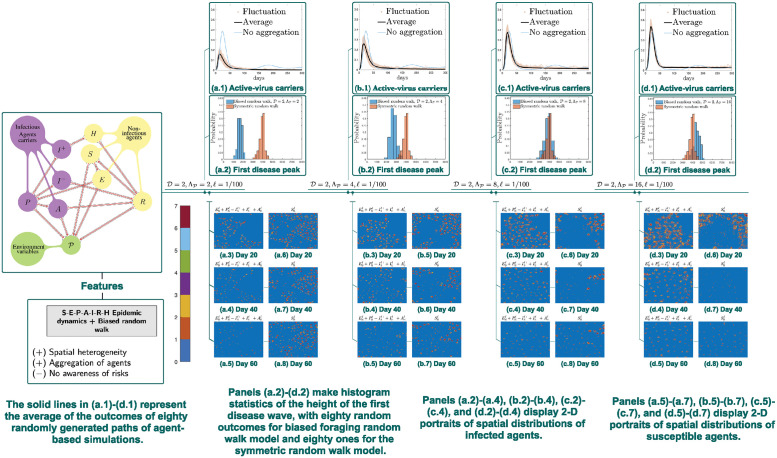
Simulations of biased random walk model. Panels (a.1)-(d.1) compare ${\bar{E}}^{\ell }(t)+{\bar{P}}^{\ell }(t)+{\bar{A}}^{\ell }(t)+{\bar{I}}^{(-),\ell }(t)+{\bar{I}}^{(+),\ell }(t)$. Increasing the spread rate $\Lambda_{P}$ of popularity variable from 2 to 4 to 8 to 16 suppresses the spatial heterogeneity, so that aggregation clusters of the susceptible decrease in number and size. As a consequence, the spread of the epidemic accelerates.

Recall that in the preliminary symmetric random walk model (Fig 2), it is always the case that the biggest outbreaks appear along with the highest random walk speed. Moreover, our expectation is that biasness of movement will lead to clustering, which prevents long-distance movements and increases spatial heterogeneity and locality, and thus suppresses disease peaks. Therefore, with the aim of investigating this suppression effect, we choose to perform simulations to study effects of directed foraging behavior under D=2. It turns out that biased random walk foraging-behavior simulations demonstrate stronger effects in deescalating the first outbreaks (Fig 4), compared with corresponding agent-based symmetric random walk simulations in Fig 2.

#### 3.2.1 Parameter values and initial data

We use the same parameters as in Table 2, with *ℓ* = 1/100, D=2, together with additional parameters concerning the popularity variable specified in Table 3 below.

**Table 3:** Event types, parameter values and corresponding physical meanings for the biased random walk model.

Event types	Parameters values	Physical meanings
Increase of P	$I_{P}=1$	parameter determining rate of Type (X) clock
	${\bar{\delta }}_{P}^{+}=0.3$	size of increment
Spread of P	$\Lambda_{P}=2,4,8,16$	parameter determining rate of Type (XI) clock
	*α* = 0.125	magnitude of spread
Decrease of P	$D_{P}=1$	parameter determining rate of Type (XII) clock
	${\bar{\delta }}_{P}^{-}=0.36$	size of decay
	$P_{\text{min}}=1/30$	minimum value of P

The initial data for each compartment are set as the same as those for simulations of the preliminary symmetric random walk model (Section 3.1.2). Additionally, to incorporate P, we assume that initially P is set to be $P_{\text{min}}=1/30$ on every site.

#### 3.2.2 Daily cases and 2D portraits of agent spatial distributions

For agent-based symmetric random walk models, spatial heterogeneity emerges in the spatial spread of the epidemic (see e.g. the spatial portraits in Fig 2); however, due to the fact that individuals move according to a symmetric random walk, the spatial distribution of agents (if we ignore the differences of their epidemiological states) remains homogeneous in expectation. In contrast, thanks to biased movement patterns, spatial heterogeneity is made stronger in the simulations here (Fig 4). Moreover, decelerating spread of the popularity information also appears to have a similar effect. Namely, spatial heterogeneity indeed becomes more evident when the spread of information decelerates (Fig 4), as spatial clusters of the susceptible agents are visually increased in number and decreased in size; at the same time, the spread of epidemic is also impeded as the infectious agents can only affect fewer of these smaller settlements.

It is also worth noting that in the biased random walk foraging model, the first wave is not only slower in speed but also smaller in its final size under a decelerated spread of P. As observed in Fig 4, whenever there is a stronger spatial heterogeneity and less macroscopic gatherings, locally clustered subpopulation can reach the state of herd immunity within themselves earlier and thus prevent the epidemic from further transmissions.

Decelerated spread of the popularity variable is associated with lower level of connectivity of population aggregations located far away. Moreover, it is well documented that epidemic spread is generally faster in urban area than rural area, e.g. in the USA (see e.g. [88, 89]). A possible explanation is that a municipal surrounding usually leads to fewer population clusters but larger in size; in contrast, slower information spread about location popularity generally gives rise to small-scale aggregations where few migrate between two settlements.

In Fig 4(a.1)–4(d.1), agent-based simulations exhibit suppressed disease peaks than simulations of the average of paths of agent-based simulations of unbiased random walk model as described in Section 2.3. Additionally, epidemic spread (till day 60) grows faster along with an increase of $\Lambda_{P}$, as displayed in Fig 4(a.5)–4(d.5), 4(a.6)–4(d.6) and 4(a.7)–4(d.7). Specifically, at each fixed time (day 20, 40, and 60), larger cavity (i.e., lower density regions of susceptible agents) appears as $\Lambda_{P}$ increases, which indicates that the first wave of the epidemic has affected a larger fraction of the total population. This is expected since Fig 4(a.1)–4(d.1) already exhibit reduced outbreak-restraining effects as $\Lambda_{P}$ increases. In the panels Fig 4(a.2)–4(a.7), 4(b.2)–4(b.7), 4(c.2)–4(c.7) and 4(d.2)–4(d.7), as the spread of information accelerates, fragmented population clusters grow to be more connected and larger in size. This may well represent the real-world phenomenon that a population with fast-spreading information (possibly associated with urbanization) is more likely to form larger aggregations, which consequently may escalate the spread of disease. Indeed, in real life, generally, rural area is paired with lower speed of spread of the popularity variable, compared with urban area. This is possibly due to lack of efficient information dissemination. Indeed it is also well observed that large-scale population mobility is relatively lower in rural area, which usually witnesses a smaller outbreak and slower spread of the epidemic than urban area.

**Remark 8** *Here we note that* Eq (3) *is no longer the corresponding equation parallel to the agent-based biased random walk foraging-behavior model described in this section. Instead, we use the agent-based simulations of the unbiased random walk model as the reference*.

### 3.3 Simulations to investigate impacts of the awareness factor

According to the biased random walk foraging-behavior model (Fig 4), weaker outbreak-flattening effects are exhibited in the presence of faster spread of the popularity variable. So it is natural to investigate impacts of the awareness variable under $D=2,\Lambda_{P}=16$, where the popularity variable spreads rapidly.

#### 3.3.1 Parameter values and Initial data

The same parameter values in Tables 2 and 3 used to create Fig 4(d.1) are going to be adopted. Here *ℓ* = 1/100, D=2 and $\Lambda_{P}=16$ are fixed. There are additional parameter values concerning the awareness variable which are specified in Table 4 below.

**Table 4:** Event types, parameter values and corresponding physical meanings for the preliminary agent-based symmetric random walk model.

Event types	Parameters values	Physical meanings
Decrease of A	$D_{A}=1$	parameter determining rate of Type (XIII) clocks
	*θ*^−^ = 0.2	percentage of decay
Spread of A	$\Lambda_{A}=20$	parameter determining rate of Type (XIV) clock
	*η*_3_ = 0.125	magnitude of spread
Increase of A	$\theta_{r}^{+}=0.2$	increment size due to hospitalization events
	$\theta_{a}^{+}=1$ , 3, 10, 20	increment size due to symptomatic cases
	$I_{A}=1$	parameter determining rate of Type (XV) clock

The initial data for agent compartments are the same as those for simulations of the biased random walk foraging-behavior model (Section 3.2.1). Moreover, to incorporate A, we assume that initially it is uniformly zero over every site, which stands for the scenario that the public has little knowledge of the epidemic at its very beginning.

#### 3.3.2 Daily cases, 2D portraits of agent distributions, and statistical histograms of peak height

**Fig 5:**
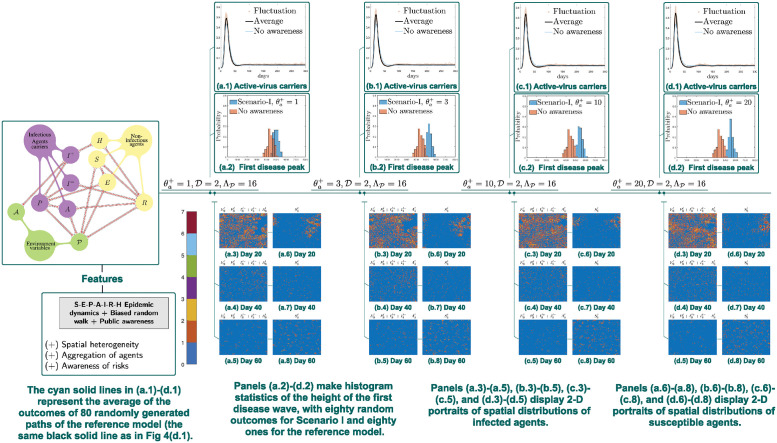
Simulations of Scenario I: Integrating awareness into biasness of random walks. Panels (a.1)-(d.1) compare ${\bar{E}}^{\ell }(t)+{\bar{P}}^{\ell }(t)+{\bar{A}}^{\ell }(t)+{\bar{I}}^{(-),\ell }(t)+{\bar{I}}^{(+),\ell }(t)$, where the reference is the biased random walk model without awareness in Section 2.4. Incorporated awareness in biasness alone does not necessarily constrain outbreaks. When the increment of public awareness $\theta_{a}^{+}$ increases from 1 to 3 to 10 to 20, there is a high chance that the susceptible agents have already transitioned into the exposed or asymptomatic infectious ones. As a result, disease transmissions are boosted and outbreaks are escalated.

**Scenario I**. In this scenario, we find that A incorporated in biasness alone does not necessarily constrain outbreaks compared with (average of multiple outcomes of) the corresponding biased random walk foraging model described as in Section 2.4. In fact, even larger outbreaks arise when individuals in our model become increasingly vigilant to signs of an ongoing outbreak, i.e., when $\theta_{a}^{+}$ increases (Fig 5). In other words, disease responses may aggravate disease outbreak if each individual responds independently upon their awareness.

But such finding actually coincides with our real-life observations. It is well documented that a trend of people traveling away from high-risk areas leads to aggravating disease outbreaks. Before moving away, there is a high chance that the agent has already been infected in the high prevalence area and will further spread the disease to places where the epidemic prevalence level is still relatively low. As a result, disease transmissions are boosted and outbreaks are escalated.

It is clearly visualized that the first disease peak can even be heightened in Scenario I, compared with the biased random walk foraging model described as in Section 2.4 (Fig 5(a.2)–5(d.2)). The same transition appears when $\theta_{a}^{+}$ increases, that is, from (a.2)-(d.2), there is a significant shift of the histogram, which also indicates an escalated peak of the first wave.

**Scenario II-i**. For Scenario II-i, the initial data and parameters will be set the same as those for simulations of Scenario I (Sections 2.5.2). Compared to Scenario I (Section 2.5.2), A in Scenario II-i displays a distinctive type of impacts, namely, the first outbreaks are delayed and reduced as the growing rate of A increases.

**Fig 6:**
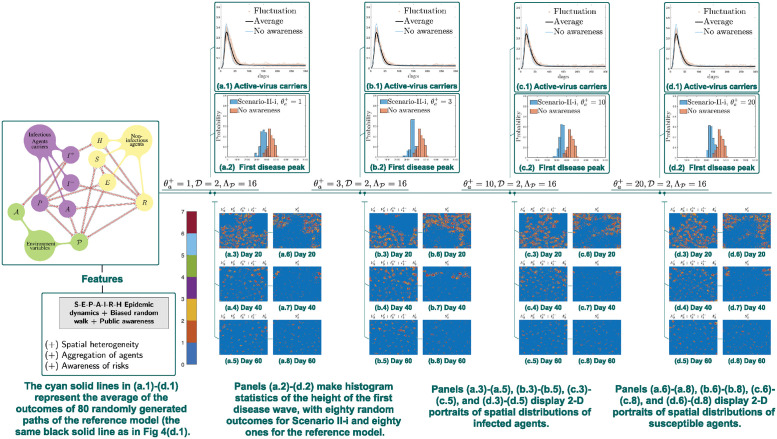
Simulations of Scenario II-i: Locally-supervised disease awareness. Panels (a.1)-(d.1) compare ${\bar{E}}^{\ell }(t)+{\bar{P}}^{\ell }(t)+{\bar{A}}^{\ell }(t)+{\bar{I}}^{(-),\ell }(t)+{\bar{I}}^{(+),\ell }(t)$, where the reference is the biased random walk model without awareness in Section 2.4. Disease control is achieved if the whole community takes response in a collaborative way. When the increment of public awareness $\theta_{a}^{+}$ increases from 1 to 3 to 10 to 20, the agent mobility is restrained. As a result, the disease peaks are flattened and an evident suppressed peak of the first wave is observed.

Fig 6(a.2)–6(d.2) provide an evidence that as $\theta_{a}^{+}$ increases, the disease peaks are flattened in Scenario II-i, as an evident shift of the histogram arises (see (a.2)-(d.2)), which also indicates a suppressed peak of the first wave. In other words, outbreaks are controlled as agent mobility is restrained. It is worth noting that an opposite type of transition patterns is displayed in Fig 5(a.2)–(d.2), where disease outbreaks are escalated rather than being controlled as $\theta_{a}^{+}$ increases. This indicates that mere aversion to disease transmission does not necessarily lead to suppression of outbreaks.

**Scenario II-ii**. In numerical simulations, Scenario II-ii displays the same but evidently stronger impact than that observed in Scenario II-i, namely, the first outbreaks are delayed and reduced as $\theta_{a}^{+}$ increases. Here the initial data and parameters are set as the same as those for simulations of Scenario I (Section 2.5.2).

**Fig 7:**
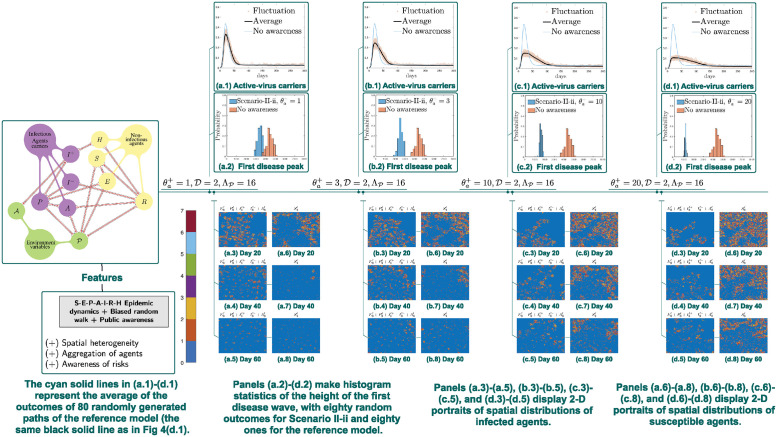
Simulations of Scenario II-ii: Spatially-uniform awareness. Panels (a.1)-(d.1) compare ${\bar{E}}^{\ell }(t)+{\bar{P}}^{\ell }(t)+{\bar{A}}^{\ell }(t)+{\bar{I}}^{(-),\ell }(t)+{\bar{I}}^{(+),\ell }(t)$, where the reference is the biased random walk model without awareness in Section 2.4. When the increment of public awareness $\theta_{a}^{+}$ increases from 1 to 3 to 10 to 20, a coordinated disease control campaign is implemented in the whole region. As a result, the first epidemic peak is are strongly delayed and reduced.

In Fig 7(a.1)–7(d.1), agent-based simulations exhibit delayed and suppressed disease peaks as $\theta_{a}^{+}$ increases. Indeed, in 2D spatial portraits of susceptible agents in Fig 7, spread of the epidemic decelerates as $\theta_{a}^{+}$ increases (till day 60). Particularly, by comparing Fig 7(a.6)–7(d.6) at day 20, it is seen that area of cavity, i.e., lower density regions of susceptible agents, decreases as $\theta_{a}^{+}$ increases. The same type of transitions is displayed in panel sets Fig 7(a.7)–7(d.7) (day 40) and Fig 7(a.8)–7(d.8) (day 60).

What’s more, it is clear that effects on disease control are stronger in Fig 7 than those in Fig 6. This implies that coordinated traffic restrictions are more helpful than localized mobility constraint prompted by disease awareness of agents on each site. As a result, we expect that a community will effectively control the outbreak only when people work together, albeit pandemics are very likely to exploit divisions among individuals. Indeed, rather than allowing each individual to respond independently upon their awareness, disease control is achieved only if the whole community takes response in a collaborative way, such as following traveling restrictions, staying-at-home orders, etc.

## 4 Conclusions and discussions

### 4.1 Conclusions

In this paper, we study the roles played by spatial and informational heterogeneities at multiple levels in a disease outbreak. Simultaneously, the interplay between such heterogeneities and various types of Public Health and Social Measures (PHSMs) has also been investigated. To highlight the major observations obtained in this study, we first note that whenever spatial heterogeneity is introduced into the system, in comparison to continuum and spatially homogeneous simulations, the corresponding agent-based simulations constantly produce suppressed and delayed disease peaks. This trend remains robust and consistent for spatial heterogeneity introduced by randomness (see Fig 2), or by incorporation of environment variables like P (Fig 4).

What’s more, disease outbreak escalations are also observed in the biased random walk foraging-behavior model (Section 2.4) whenever $\Lambda_{P}$, the speed of spread of information about location popularity P, increases (Fig 4). Moreover, we note that there is a subtle difference between impacts of D and $\Lambda_{P}$ to the first disease wave. Namely, increased $\Lambda_{P}$ not only leads to an earlier peaking time but also a larger final size of the first disease wave. Indeed, with increased macroscopic clustering, there is less suppression to the spreading of epidemic between two clusters, and thus the state of herd immunity can only be reached macroscopically when the epidemic has reached all the subpopulations. At the same time, smaller clustering, which arises with a decreased $\Lambda_{P}$, leads to a herd immunity early enough such that active-virus carriers have not yet escaped to somewhere else. This also coincides with the real-world scenario that a decreased D represents travel restrictions, while a decreased $\Lambda_{P}$ resembles a relatively more strict policy such as stay-at-home orders.

As for the awareness factor A, we demonstrate that public knowledge and aversion of the disease can have notably distinct impacts, depending on the strategy the community adopts in response to the outbreak (Section 2.5). The first type of strategies we assess lead to individualized actions to avoid places with visibly high prevalence. This is discussed in Scenario I (Section 2.5.2), where A is configured to only affect the direction of agent movement. What we observe is that this strategy do not automatically bring about disease control effects, when compared to the biased random walk foraging model that do not incorporate the A factor as described in Section 2.4. In fact, negative impacts can even be made to epidemics spread, like escalated disease peaks, if population mobility is not effectively controlled (Fig 5).

Nevertheless, there is a strong indication that disease response measures that can make the whole population or a part of it to be less mobile is helpful in fighting disease transmissions. This type of disease control effects is observed in Scenario II, including Scenario II-i (Section 2.5.3), which assumes reduced population mobility based on awareness of local disease transmissions (Fig 6), as well as in Scenario II-ii, where a centralized disease control measure is additionally implemented (Fig 7). Furthermore, our simulations strongly indicate that in comparison to Scenario II-i, Scenario II-ii is significantly more effective in suppressing the first wave of the outbreak. This suggests that a coordinated disease censoring and control system can be vital in the response of a newly emerging pandemic. To summarize, the mere presence of individual awareness and desire to avoid the disease will not automatically ensure the suppression of the outbreak at the community level. Fortunately, an effective control can be achieved when we unite and respond to the outbreak wisely.

When comparing our finding to existing literatures, we can see that very different modeling methodologies may result in highly correlated epidemiological insights, which further supports the robustness of these findings. Firstly, in [30], leveraging the recognition in ecological studies that the spatial distribution of natural populations consists of subpopulations instead of being homogeneous, models of dynamics of infectious diseases with the existence of subpopulations of hosts as a consequence of spatial partitioning are studied; results indicate that when the degree of spatial partition decreases, and when time of visitation between localities increases (through which the contact between subpopulations is achieved), the transmission of disease becomes more prone to occur, which is in alignment with our results that addition of spatial heterogeneity introduced by randomness results in de-escalated severeness of the first episode of the disease outbreak in agent-based symmetric random walk model compared with the corresponding continuum simulations (Fig 2 in Section 3.1.3).

Secondly, using real-life data, copious studies have confirmed that outbreaks of infectious disease generally display clear spatial patterns with complex geographies, such as a high level of regional disparities and fragmentations (see e.g. [22], [23], [26], [21] and [20]). This coincides with our main proposals in this article, that it may be beneficial to understand disease outbreaks as socio-spatial processes with heterogeneity, and it is highly important to carry out systematic investigations of formation of epidemic spatio-temporal patterns. Particularly, in [28], a stochastic SIR model assuming homogeneous mixing turns out to fail to capture certain specific feature of disease wave duration, compared with the results of an individual-based SIR model set over a discrete lattice with foraging-like host movements for heterogeneously distributed resource. We note that as the focus of study of [28] is on animal hosts, agent movement behavior is assumed to be driven by resources like food, water, etc. In contrast, humans are more likely to make use of information (for example the popularity level of locations) to direct their social behavior as well as make responses to the prevalence level of the disease progression.

Moreover, [25] builds a computational georeferenced metapopulation approach consisting of various data layers for capturing the transportation infrastructures and mobility patterns. It is observed that whenever the radiation pattern of epidemic to the neighboring areas is reminiscent of the process of diffusion, a stronger correlation arises in the evolution of the pandemic at the local level. The synchronization of the local epidemic profiles is enhanced by addition of the dynamics of local commuting (Fig 3 in [25]). Additionally in [31], they discover that, for a given prevalence at the system level, the higher degree of mixing between individuals and subpopulations (which in the context of [31] arises due to Markovian approaches in a memoryless model) leads to much faster occurrences of the epidemic progression, reaching a much larger fraction of subpopulations. These aforementioned findings are in agreement with our result that increasing D, the rate of movement, in the agent-based model leads to a system whose dynamics is closer to the ODE, producing heightened disease peaks.

Furthermore, in [23], an analysis of German COVID-19 outbreak (2020) is conducted, where they find that the most important reason for the rapid spread of COVID-19 in February 2020 in Heinsberg district is arguably the carnival celebration coinciding with the disease onset there. Compared with at least 390 cases related to this outbreak, another super-spreading event occurring in a birthday party in a nightclub in Berlin leads to a much less prominent outbreak, with only 53 related infections. The reason of this gap is discussed: the carnival festivities in Heinsberg attracted a relatively larger audience from much farther away regions. Indeed, this discovery of [23] lends support to our result that increased $\Lambda_{P}$, i.e., increased spread speed of popularity, leads to an increased final size of the first disease wave. What’s more, they argue that returnee infections cases of individuals returning from touristic or business trips are shown to be an example of significant relocation diffusion. According to the specific data sample in [23], the impact of tourist mobility is demonstrated to be extremely widespread spatially. This result is in agreement with our findings about disease response strategy of Scenario I which does not necessarily lead to disease control, where individuals leaving high prevalence sites may prompt the spread of epidemic to communities not yet hit by the outbreak. Another article that supports our discovery about effects of disease awareness and responses is [90]. Indeed, it is pointed out in [90] that in many parts of the world, spatially heterogeneous COVID-19 curves with an absence of attention to potential of epidemic spatial spillovers may be the result of a fragmented public health response; the patchwork of health interventions whose focus has been on interruptions of transmission within but not across regional boundaries has resulted in case resurgences in areas that successfully flattened the epidemic curve beforehand. Therefore, the conclusion of [90] is in line with our finding about helpfulness of a coordinated disease response. We recall that Scenario II-ii, the response strategy with coordinated and centralized disease censoring system, is considerably more effective in controlling disease transmission compared to Scenario II-i, where agent mobility is assumed to decrease based on local awareness of epidemic severeness level.

### 4.2 Mathematical understanding of the spatial correlation

It is worth noting that the correlation of interaction discussed in this study is also intrinsically connected to the finite range effect, the celebrated mathematical theories of contact process as an Interacting Particle Systems (IPS) ([91] [92] [93]). The contact process can be seen as an agent based SIS model on *Z*^*d*^ (or other infinite graphs) where each site resides a non-moving agent. An infected agent with rate λ passes the pathogen to someone randomly chosen from a certain neighborhood of them, and with rate 1 recovers and gets back to State S. A key characteristic of such a system is that, there is a critical value λ_*c*_ > 1 such that the epidemic can have a positive probability to survive if and only if λ > λ_*c*_ [94]. In other words, when λ ∈ (1, λ_*c*_], although we seem to have *R*_0_ > 1 and the corresponding mean-field ODE survives, the locality, which is now in the sense of interaction range rather than moving speed, may also impede disease transmission and force the stochastic system to remain sub-critical. Meanwhile, as the range of interaction goes to infinity or, when there is a mechanism of fast stirring (neighboring sites exchanging their values, like moving), the contact process will also enjoy increasing homogeneity and converges in distribution to its mean-field model or have λ_*c*_ → 1 [94, 95]. In our study, on the other hand, we have been focusing more on the case when the transmission rate is given to be super-critical and explore how the interactions of agent activity and information dynamics affect the size and severity of the pandemic.

### 4.3 Future works

As for future works, firstly, we would like to find suitable ways for modeling of more than one single variant of concern (VOC). There are many real-life scenario that are related with dynamics of multiple VOCs. For example, suppose that when an epidemic is already peaked and enters the stable state, becoming a so-called “endemic”, a new VOC emerges. Then an interesting question to ask is how long does it take for the new VOC to make the second wave to take place.

Secondly, we are interested in introducing medical resource limitations into the system, which is not yet considered in our model in this paper. For example, with hospital capacity strain, with COVID, death toll has turned out to be catastrophic. For now, A is assumed to change according to two things: visible infectious case numbers and hospitalization events. It will be of interest to also include medical system occupation ratio as a driven factor of the awareness level and thus the implementation of stronger PHSMs. In this case it is possible to observe positive effects of public awareness to hospital-overload prevention.

Moreover, besides Non-pharmaceutical Interventions (NPIs) that are considered here, pharmacological interventions can also be incorporated into the system, such as vaccinations, medications, etc. With limited medication supplies, how to optimize their distributions among the whole population can be studied after this. Apparently one factor is type of effects of the medication. For example, currently COVID medications mainly target those who develop severe symptoms, which may lead to alleviation of pressure for hospital beds.

Finally, we remark that the evolution of popularity and awareness as information field should be continuous in essence, even though the models proposed in this study is mostly discrete in space and our findings make us believe that the discrete nature of agents is crucial in capturing the finiteness of correlation and heterogeneity in disease transmission. Thus it should be more suitable to consider these environment variables to be updated in a continuum manner through kinetic modeling. Moreover, we note that there are quite a few works taking into account of spatial heterogeneity in the context of kinetic modeling, on the mesoscopic scale. Particularly, a kinetic model of crowd dynamics, which is a relatively more classical topic (see e.g. [45, 47, 48, 53]), is adopted as a base. Then an epidemic contagion process is coupled with the crowd dynamics model. These models can be built on network [96], or the continuum space of space dimension one or two [46]. It is worth mentioning that a multi-scale model encompassing miscro-scopic, mesoscopic, and macro-scopic scales in an aligned way is first introduced in [44]. In this way, evolutions of the virus itself and cells as well as individuals and further up to the collective behavior of populations are all taken into consideration. Along this line there are a number of works emerging recently, e.g. [49, 51, 52, 97]. Inspired by the works in [98], in subsequent studies we are going to modify the model in this paper into a multi-scale framework. While keeping the discrete nature of agents as well as their epidemiological dynamics, one may allow the popularity and awareness level to be continuous in time and space, and let them evolve as a Piecewise-Deterministic Markov Process, i.e., the fields of information follow their PDEs until they are interrupted by random transitions at the epidemiology level. Then the PDEs restart with updated parameters and (or) initials conditions.

## Supporting information

S1 TextThis file contains supplementary figures for sensitivity analysis and further discussions.**Fig A**: Simulations of symmetric random walk model with various values of Ds’ and *δ*_*R*_ = 0. **Fig B**: Simulations of biased random walk model with more different combinations of D and Λ_*P*_. **Fig C**: configurations of mobile population in the simulations of biased random walk model. **Fig D**: Simulations of biased random walk model under different values of the scaling factor. **Fig E**: configurations of active virus carriers in simulations of biased random walk model under different values of the scaling factor. **Fig F**: Simulations of the scenario when awareness level reduces the rate of infectious contacts.(PDF)

S1 Data Records of simulation results which can be used to replicate study findings reported in this paper. (ZIP)

S2 Data MATLAB sources code package and records of simulation results which can be used to replicate study findings reported in this paper. (ZIP)
