# Supplementary material for: Interacting particle models on the impact of spatially heterogeneous human behavioral factors on dynamics of infectious diseases
Source: PLoS Comput Biol. 2024 Aug 8;20(8):e1012345. doi: 10.1371/journal.pcbi.1012345 (PMC11335169; doi:10.1371/journal.pcbi.1012345)
Supplement: S1 Text — Fig A: Simulations of symmetric random walk model with various values of Ds’ and δR = 0. Fig B: Simulations of biased random walk model with more different combinations of D and ΛP. Fig C: configurations of mobile population in the simulations of biased random walk model. Fig D: Simulations of biased random walk model under different values of the scaling factor. Fig E: configurations of active virus carriers in simulations of biased random walk model under different values of the scaling factor. Fig F: Simulations of the scenario when awareness level reduces the rate of infectious contacts. (PDF) [file pcbi.1012345.s001.pdf]

Supplementary Material of article 'Interacting particle models on the impact of spatially heterogeneous human behavioral factors on dynamics of infectious diseases'

Yunfeng Xiong[1], Chuntian Wang[2], Yuan Zhang[3*],

1 School of Mathematical Sciences, Beijing Normal University, Beijing, China
2 Department of Mathematics, The University of Alabama, Tuscaloosa, Alabama, United States of America
3 Center for Applied Statistics and School of Statistics, Renmin University of China, Bejing, China

These authors contributed equally to this work.
* zhang_probab@ruc.edu.cn

Abstract

In this supplementary note, we provide additional numerical simulations to support our investigations in the main text. This note is organized as follows. Firstly, we will study the scenario where immunity will never wane. Secondly, we will carry out further investigations to agent spatial pattern formations under different mobility speed of agent and spread rate of the popularity variable. Thirdly, we make a sensitivity analysis of the temporal scaling parameter between environment variables to show robustness of our epidemiological model. Finally, we investigate a new type of scenario by integrating public awareness upon infectious contacts. Under this scenario, the increase of public-awareness increasing rate directly suppresses the contact rate of agents and thus the epidemic curve is flatten.

Contents

A Agent-based symmetric random walks without immunity waning **2**
A.1 Numerical simulations 2

B Further exploration of impacts of human natural behavior **3**
B.1 Numerical simulations 3

C Sensitivity analysis of the temporal scaling factor **4**
C.1 Numerical simulations 5

D A new scenario of integrating public awareness upon infectious contacts **6**
D.1 Impact of the awareness factor in Scenario III 7
D.2 Numerical simulations 7
D.2.1 Daily cases, 2D portraits of agent distributions, and statistical histograms of peak height 8

A Agent-based symmetric random walks without immunity waning

Throughout the article, we have taken waning of immunity into account to make the model more physical (Section 2.3.2 in the main text). But at the same time, because of this assumption, the agents who get infected in the first wave will possibly transit back to the state of S. As a result, in the 2D portraits of spatial distributions of susceptibles displayed in the main text (Fig 2 in the main text), the lower-density cavities that are created by the first wave may gradually be refilled due to spontaneous transitioning of recovered agents back to the S state. Thus it is meaningful to verify whether the area of cavities that are observed in our 2D agent-spatial-distribution portraits does indeed reflect cumulative scale of first waves. To this end, we carry out a sensitivity analysis in absence of immunity waning.

Based on the assumptions for the preliminary modeling of agent-based symmetric random walks (Section 2.3.2 in the main text), we instead assume that $\delta_R = 0$, so that the R agents will forever stay in this state and never transit to the S state. Nevertheless, as exhibited in numerical simulations (see Fig A below), only negligible differences arise on transition patterns under different human mobility rate $\mathcal{D}$. This coincides with our expectation as the speed of immunity waning is on the scale of months or even years, which is much slower than the occurence of infection, hospitalization, and recovery.

In summary, immunity waning and re-infection play an negligible role in the first 60 days of the pandemic (see Fig 2(a.1)-2(d.1) of in the main text. Thus it is legitimate to use the outflow from compartment S as an indicator of the size of the first wave in our main text.

A.1 Numerical simulations

Here we carry out the numerical experiments in order to validate that the absence of immunity waning does not change area of 'cavity' (lower density regions of susceptible agents) as observed in e.g. Fig 2(a.5)-2(a.7), 2(b.5)-2(b.7), 2(c.5)-2(c.7), and 2(d.5)-2(d.7) in Section 3.1.3 of the main text.

Below in Fig A(a.1)-A(d.1) we display time evolutions of $\bar{E}^{\ell}(t) + \bar{P}^{\ell}(t) + \bar{A}^{\ell}(t) + \bar{I}^{(-),\ell}(t) + \bar{I}^{(+),\ell}(t)$ and $\bar{E}(t) + \bar{P}(t) + \bar{A}(t) + \bar{I}^{(-)}(t) + \bar{I}^{(+)}(t)$ for agent-based simulations of the symmetric random walk model and continuum simulations for the ODEs as in Eq. 3 in the main text, respectively. The solid lines in (a.1), (b.1), (c.1), and (d.1) represent the average of the outcomes of eighty randomly generated paths of agent-based simulations. The parameter values and initial data are set as the same as those used to create plots in Fig 2(a.1)-2(d.1) in the main text, with the only exception that $\delta_R = 0$. Particularly, Fig A(a.1)-A(d.1) are parallel with Fig 2(a.1)-2(d.1) in the main text, respectively. In Fig A(a.1)-A(d.1), $\mathcal{D}$ is increased from 0.25 to 0.5 to 1 to 2.

We also display two-dimensional portraits of spatial distribution $S^{\ell}_{\mathbf{s}}(t)$ (see Fig A(a.5)-A(a.7), A(b.5)-A(b.7), A(c.5)-A(c.7), and A(d.5)-A(d.7)), and $E^{\ell}_{\mathbf{s}}(t) + P^{\ell}_{\mathbf{s}}(t) + A^{\ell}_{\mathbf{s}}(t) + I^{(-),\ell}_{\mathbf{s}}(t) + I^{(+),\ell}_{\mathbf{s}}(t)$ (the remaining panels of Fig A). Panels (a.2)-(a.7) show time slices (at day 20, 40, and 60) associated with one random path of the agent-based simulations displayed in Fig A(a.1), and the same also applies to (b.2)-(b.7), and (b.1); and (c.2)-(c.7), and (c.1); and (d.2)-(d.7), and (d.1); and Particularly, Fig A(a.2)-A(a.7), A(b.2)-A(b.7), A(c.2)-A(c.7), and A(d.2)-A(d.7) are parallel with Fig 2(a.2)-2(a.7), 2(b.2)-2(b.7), 2(c.2)-2(c.7), and 2(d.2)-2(d.7) in the main text, respectively.

Comparing Fig A and Fig 2 in the main text, they demonstrates very similar patterns with the disease curve. The size of cavity, i.e. lower density regions of

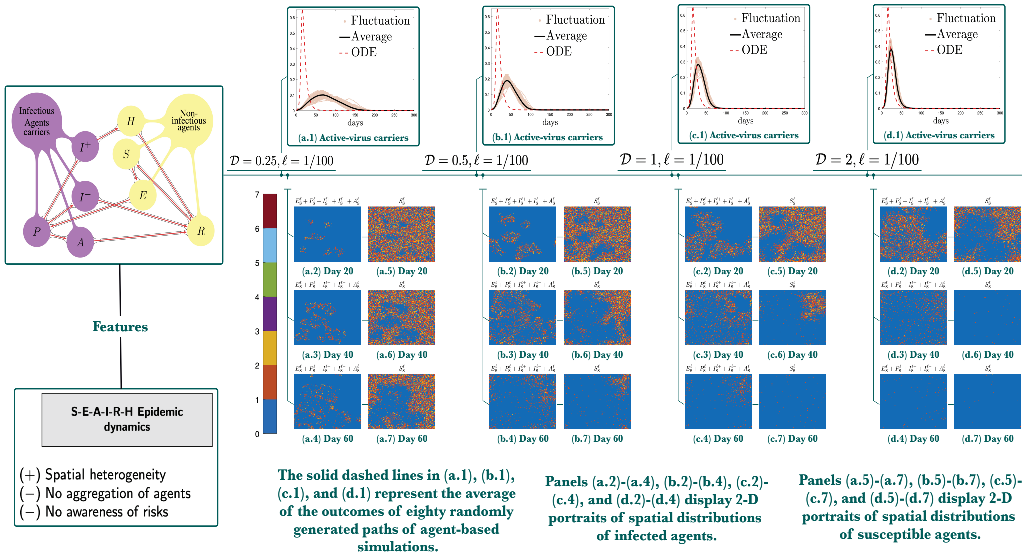

Fig A. Simulations of symmetric random walk model. Panels (a.1)-(d.1) compare $\bar{E}^{\ell}(t) + \bar{P}^{\ell}(t) + \bar{A}^{\ell}(t) + \bar{I}^{(-),\ell}(t) + \bar{I}^{(+),\ell}(t)$. Increasing the rate $\mathcal{D}$ of agent movement from 0.25 to 0.5 to 1 to 2 leads to a heightened disease outbreak, and thus escalate severeness of the first episode of the epidemic. The parameter values and initial data are set as the same as those used to create plots in Fig 2 in the main text, with the only exception that $\delta_R = 0$. Particularly, (a.1)-(d.1), (a.2)-(a.7), (b.2)-(b.7), (c.2)-(c.7), and (d.2)-(d.7) are parallel with Fig 2(a.1)-2(d.1), 2(a.2)-2(a.7), 2(b.2)-2(b.7), 2(c.2)-2(c.7), and 2(d.2)-2(d.7) in the main text, respectively.

susceptible agents, is almost the same as displayed in the panels of Fig A and Fig 2 in the main text.

B Further exploration of impacts of human natural behavior

We further explore how the interplay between human mobility and information spread speed may affect the course of outbreaks in our biased random walk foraging-behavior model constructed in Section 2.4 in the main text. Figs B and C below serve as a supplement of the numerical experiments in Fig 4 in the main text.

It is demonstrated that spatial clustering emerges under either higher agent mobility or lower spread speed of location popularity information, and epidemic curve tends to be much more flatted, as the first wave is not only slower in speed but also smaller in its final size. This again confirms our conclusion that the spatial clustering of agents impedes the contacting and consequently mitigates the epidemic outbreak.

B.1 Numerical simulations

We adopt the same initial data and parameters (except for $\mathcal{D}$ and $\Lambda_{\mathcal{P}}$) as those used to create plots in Fig 4(a.1)-4(d.1) in the main text, and simulate two additional groups with the parameter combiations of $\mathcal{D} = 0.25, \Lambda_{\mathcal{P}} = 2$ and $\mathcal{D} = 0.25, \Lambda_{\mathcal{P}} = 16$. For each group, we perform eighty independent random realizations. We note that Fig B(a) and B(b) are the same as Fig 4(d.1) and 4(a.1) in the main text, respectively.

Particularly, in Fig B, evolutions of $\bar{E}^{\ell}(t) + \bar{P}^{\ell}(t) + \bar{A}^{\ell}(t) + \bar{I}^{(-),\ell}(t) + \bar{I}^{(+),\ell}(t)$ of the agent-based biased-random-walk foraging model described as in Section 2.4 in the

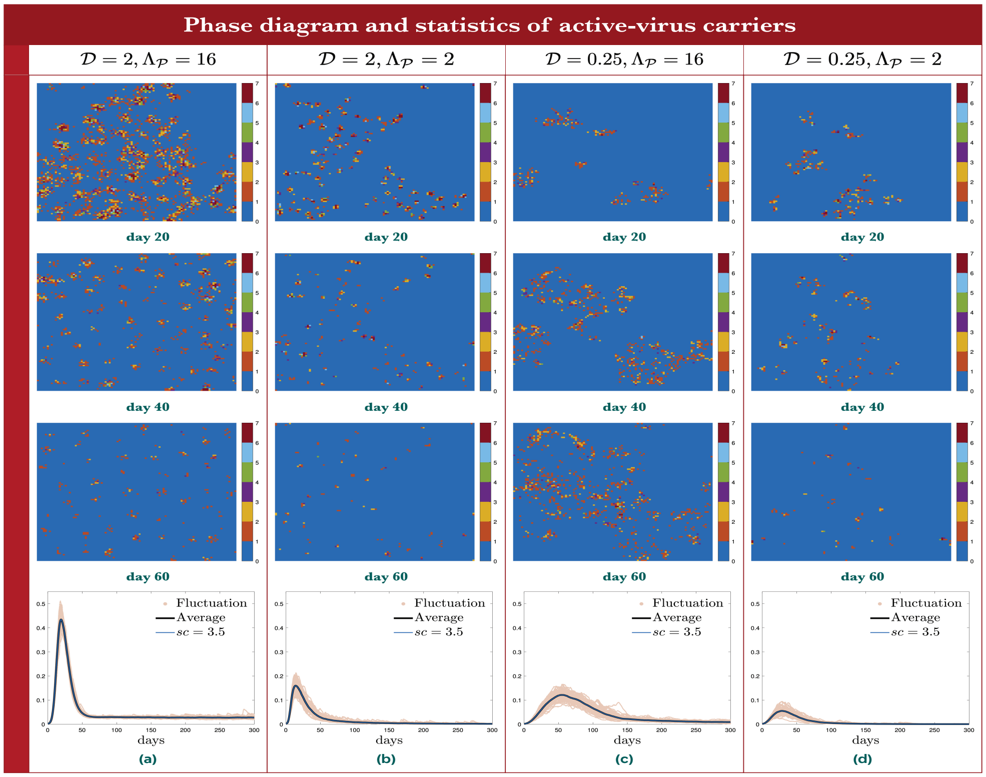

Fig B. Simulations of biased random walk model. Panels (a)-(d) compare $\bar{E}^{\ell}(t)+\bar{P}^{\ell}(t)+\bar{A}^{\ell}(t)+\bar{I}^{(-),\ell}(t)+\bar{I}^{(+),\ell}(t)$. The spatial clustering emerges under either higher agent mobility or lower spread of location popularity information, and epidemic curve tends to be much more flatted. Here (a) and (d) are the same as Fig 4(d.1) and 4(a.1) in the main text, respectively. In all the panels, ℓ is fixed as 1/100.

main text, as well as $\bar{E}(t)+\bar{P}(t)+\bar{A}(t)+\bar{I}^{(-)}(t)+\bar{I}^{(+)}(t)$ of continuum simulations for the ODEs as in Eq. 3 in the main text, are exhibited (Fig B(a)-B(d)). The remaining panels show the corresponding 2-D portraits of spatial distributions of infected agents $\left(E_{\mathbf{s}}^{\ell}(t)+P_{\mathbf{s}}^{\ell}(t)+A_{\mathbf{s}}^{\ell}(t)+I_{\mathbf{s}}^{(-),\ell}(t)+I_{\mathbf{s}}^{(+),\ell}(t)\right)$ in one realization. Moreover, 2-D portraits of spatial distributions of mobile agents $S_{\mathbf{s}}^{\ell}(t)+E_{\mathbf{s}}^{\ell}(t)+P_{\mathbf{s}}^{\ell}(t)+A_{\mathbf{s}}^{\ell}(t)+I_{\mathbf{s}}^{(-),\ell}(t)+I_{\mathbf{s}}^{(+),\ell}(t)+R_{\mathbf{s}}^{\ell}(t)$ in the same realization are exhibited in Fig C. Here $\ell=1/100$ is fixed.

Fig B clearly shows that the first disease wave can be heightened and propelled earlier in time when either $\Lambda_{\mathcal{P}}$ increases or $\mathcal{D}$ increases, or when both of them increase simultaneously. Also, in these cases, an early-die-out outbreak may have a high risk of developing into a pandemic. This type of transitions are demonstrated in each column of Figs B and C.

C Sensitivity analysis of the temporal scaling factor

In all the numerical experiments performed in the main text, we always choose the temporal scaling factor sc to be 3.5 (see Section 2.3.2 in the main text). We will demonstrate the robustness of our simulations against the choice of sc, that is, the temporal scaling dose not cause significant differences in the spatial characteristics of our biased random walk model.

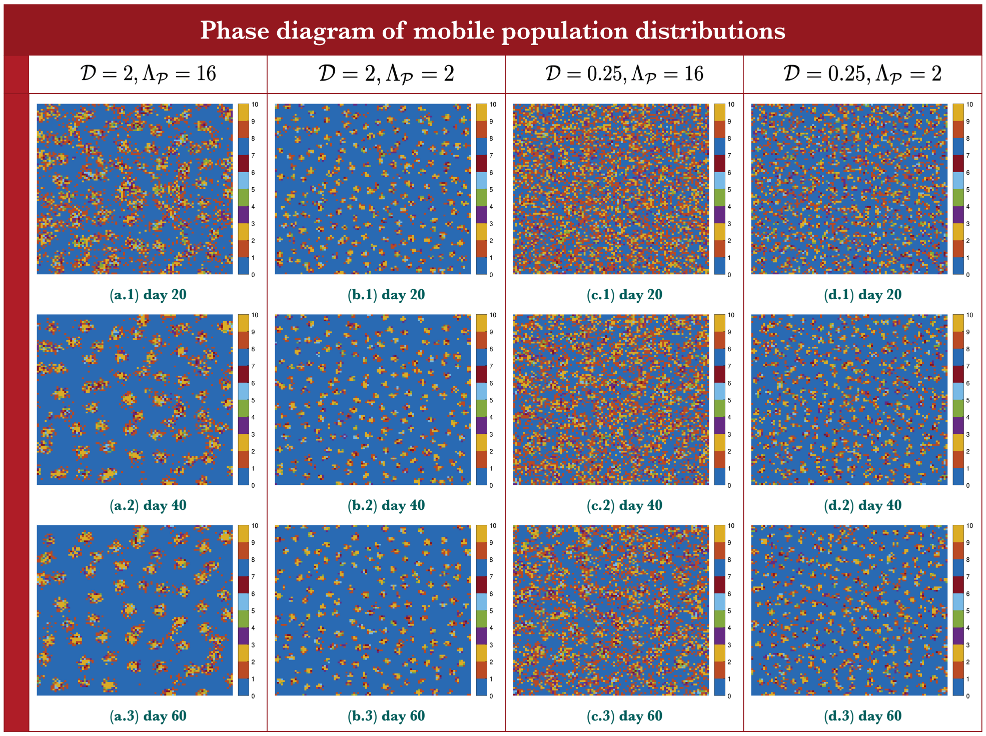

Fig C. Phase diagram of mobile population $S_{\mathbf{s}}^{\ell}(t) + E_{\mathbf{s}}^{\ell}(t) + P_{\mathbf{s}}^{\ell}(t) + A_{\mathbf{s}}^{\ell}(t) + I_{\mathbf{s}}^{(-),\ell}(t) + I_{\mathbf{s}}^{(+),\ell}(t) + R_{\mathbf{s}}^{\ell}(t)$ at day 20, 40, and 60 for agent-based simulations under different scaling factor. In all the panels, ℓ is fixed as 1/100.

When the value of sc is changed, so is the relative time scale between impacts of incorporation of $\mathcal{P}$ and $\mathcal{A}$, and physical attributes of the epidemic. Thus it is meaningful to conduct a sensitivity analysis on different values of sc.

C.1 Numerical simulations

In this section, we carry out sensitivity analysis mentioned in Section 2.3.2 in the main text on changing the value of our temporal scaling factor sc. In Figs D and E, we demonstrate evolutions of $\bar{E}^{\ell}(t) + \bar{P}^{\ell}(t) + \bar{A}^{\ell}(t) + \bar{I}^{(-),\ell}(t) + \bar{I}^{(+),\ell}(t)$, and the phase diagram of infected agents $E_{\mathbf{s}}^{\ell}(t) + P_{\mathbf{s}}^{\ell}(t) + A_{\mathbf{s}}^{\ell}(t) + I_{\mathbf{s}}^{(-),\ell}(t) + I_{\mathbf{s}}^{(+),\ell}(t)$ at day 60 and respectively, under different temporal scaling factor sc.

All of the simulations are based on simulations of the biased-random-walk foraging model described as in Section B (also see Section 2.4 in the main text), where four groups with the parameter combinations $\mathcal{D} = 2, 0.25$, $\Lambda_{\mathcal{P}} = 2, 16$ are considered, each of which is paired with different temporal scaling factors $sc = 2$, 3, 3.5, and 5 (16 groups in total).

Fig D compares the time evolutions of $\bar{E}^{\ell}(t) + \bar{P}^{\ell}(t) + \bar{A}^{\ell}(t) + \bar{I}^{(-),\ell}(t) + \bar{I}^{(+),\ell}(t)$ for varying sc, under different choices of $\mathcal{D}$ and $\Lambda_{\mathcal{P}}$. In all the panels, the cyan solid line represents the average of the outcomes of eighty randomly generated paths of simulations with $sc = 3.5$. Despite distinct values of sc, the same transition patterns are displayed in Fig D as in Fig B, as well as in Fig 4 in the main text, which indicates that the proposed model is robust against the varying values of the scaling factor.

For the purpose of comparisons, we also display two-dimensional portraits of spatial

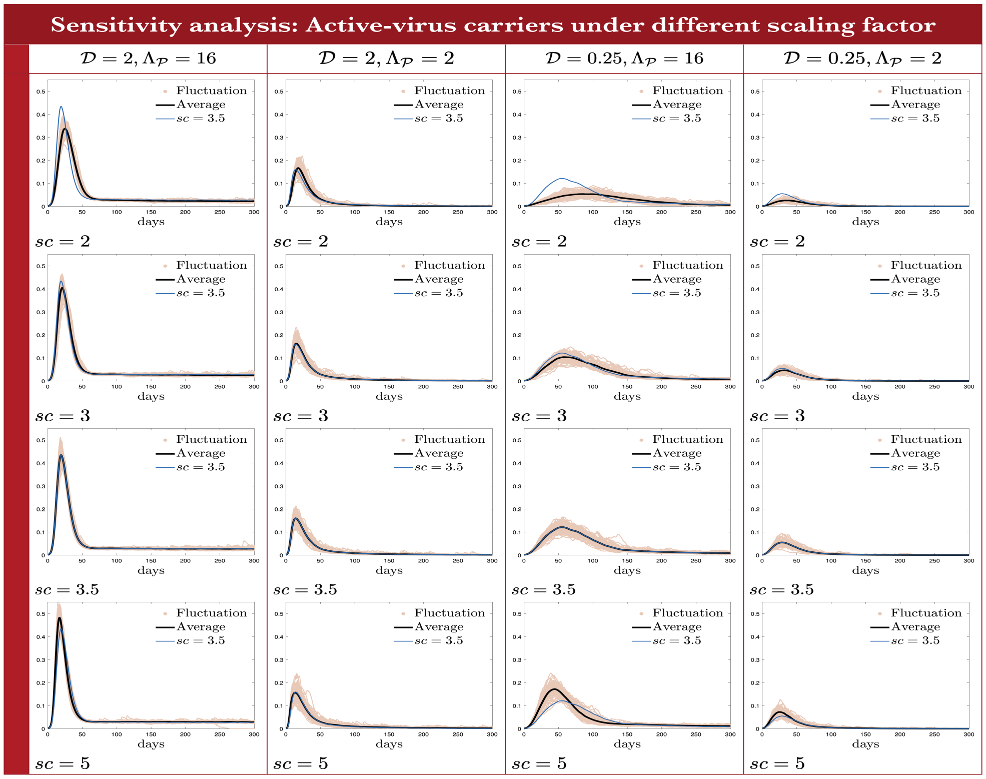

Fig D. Output of time evolutions of $\bar{E}^{\ell}(t) + \bar{P}^{\ell}(t) + \bar{A}^{\ell}(t) + \bar{I}^{(-),\ell}(t) + \bar{I}^{(+),\ell}(t)$ for agent-based simulations under different scaling factor. In all the panels, the cyan solid line represents the average of the outcomes of eighty randomly generated paths of simulations under $sc = 3.5$. The first disease wave can be heightened and propelled earlier in time when either $\Lambda_{\mathcal{P}}$ increases or $\mathcal{D}$ increases, or when both increase simultaneously. This shows the same transition patterns are displayed as in Fig B, as well as Fig 4 in the main text.

distribution of infected agents $E^{\ell}_{\mathbf{s}}(t) + P^{\ell}_{\mathbf{s}}(t) + A^{\ell}_{\mathbf{s}}(t) + I^{(-),\ell}_{\mathbf{s}}(t) + I^{(+),\ell}_{\mathbf{s}}(t)$ in one realization at day 60 in Fig E. It is shown that the patterns of spatial clustering are almost the same under different temporal scaling.

D A new scenario of integrating public awareness upon infectious contacts

Rather than assuming that agent movement patterns depend upon the awareness variable $\mathcal{A}$ as in Section 2.5 in the main text, we consider a different scenario, called Scenario III subsequently, where it is the rate of epidemic transmissions that changes according to $\mathcal{A}$. This scenario corresponds to the public response strategy to reduce infectious contacts, such as mask mandates, social distancing, etc. As for the dynamics of $\mathcal{A}$, we assume that it is the same as in Scenario I (Section 2.5.2 in the main text). What we observe below through simulations of Scenario III (see Fig F below) is a substantial suppression of the initial disease wave when compared to the corresponding biased random walk foraging model with an absence of the $\mathcal{A}$ factor (Section 2.4 in the main text).

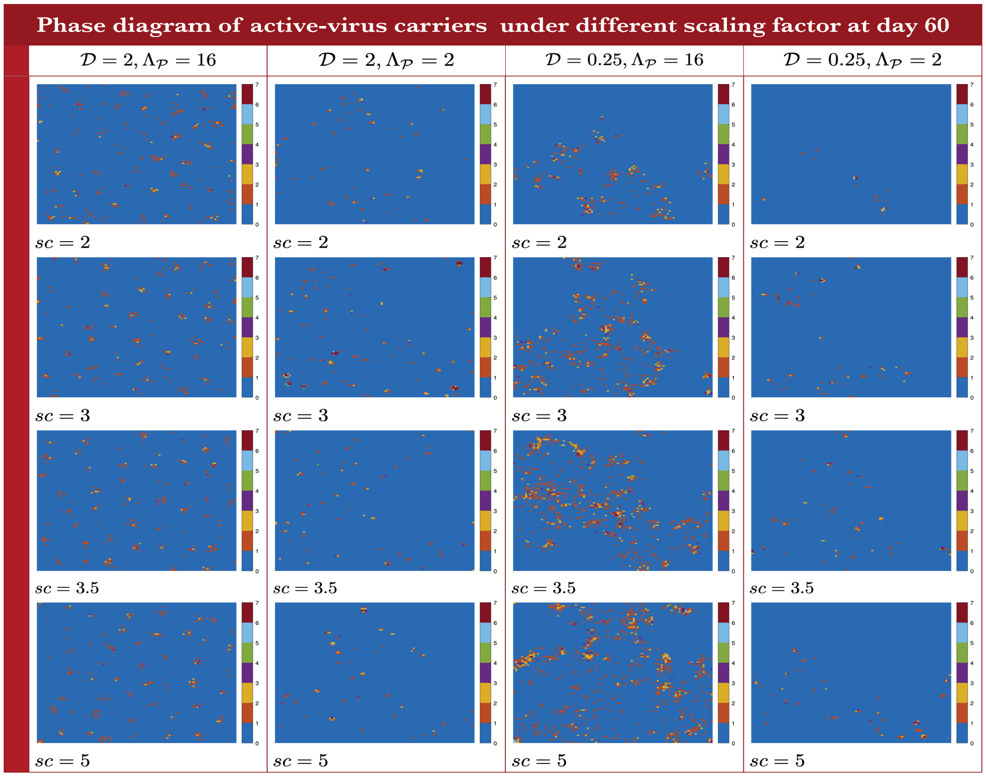

Fig E. Phase diagram of $E^{\ell}_{\mathbf{s}}(t) + P^{\ell}_{\mathbf{s}}(t) + A^{\ell}_{\mathbf{s}}(t) + I^{(-),\ell}_{\mathbf{s}}(t) + I^{(+),\ell}_{\mathbf{s}}(t)$ at day 60 for agent-based simulations under different values of scaling factor. The patterns of spatial clustering are almost the same under different temporal scaling. This shows that the same patterns are displayed as in Fig B, as well as Fig 4 in the main text.

D.1 Impact of the awareness factor in Scenario III

During outbreaks of diseases, public awareness may be aroused, causing healthy individuals to reduce contacts consciously in order to avoid transmissions. This is especially the case when there are public health and social measures such as a mask mandates on public transportation, and the practice of social distancing. Note that in comparison with travel restrictions which reduce spatial movement, the aforementioned measures can be effective even for individuals residing at the same site. Motivated by this real-world scenario, we assume that infectious contacts occur with probability $\left(1 - \mathcal{A}^{\ell}_{\mathbf{s}}(t)\right)$ upon an advancement of Type (II) Poisson clocks described in Section 2.3.2 in the main text, where this probability is set as one in the absence of the awareness factor.

D.2 Numerical simulations

Below in Fig F(a.1)-F(d.1), we display time evolutions of $\bar{E}^{\ell}(t) + \bar{P}^{\ell}(t) + \bar{A}^{\ell}(t) + \bar{I}^{(-),\ell}(t) + \bar{I}^{(+),\ell}(t)$ of simulations of Scenario III. The black solid lines represent the average of the outcomes of eighty randomly generated paths of agent-based simulations. The parameter values and initial data are set as the same as those used to create plots in Fig 5(a.1)-5(d.1) in the main text. Particularly, Fig F(a.1)-F(d.1) are parallel with Fig 5(a.1)-5(d.1) in the main text, respectively. In Fig F(a.1)-F(d.1), θ^{+}_{a} increases from 1 to 3 to 10 to 20. The biased random walk foraging

model without awareness in Section 2.4 in the main text is chosen as the reference. Comparisons are made bewteen $\bar{E}^{\ell}(t)+\bar{P}^{\ell}(t)+\bar{A}^{\ell}(t)+\bar{I}^{(-),\ell}(t)+\bar{I}^{(+),\ell}(t)$ by eighty random paths of simulations of Scenario III and the average of eighty random outcomes of the reference model (the same black solid as in Fig 4(d.1) in the main text) represented by the cyan solid lines. We also display two-dimensional portraits of spatial distribution $S^{\ell}_{\mathbf{s}}(t)$ (Fig F(a.5)-F(a.7), F(b.5)-F(b.7), F(c.5)-F(c.7), and F(d.5)-F(d.7)), and $E^{\ell}_{\mathbf{s}}(t)+P^{\ell}_{\mathbf{s}}(t)+A^{\ell}_{\mathbf{s}}(t)+I^{(-),\ell}_{\mathbf{s}}(t)+I^{(+),\ell}_{\mathbf{s}}(t)$ (the remaining panels of Fig F).

D.2.1 Daily cases, 2D portraits of agent distributions, and statistical histograms of peak height

Through simulations of Scenario III, we find that as individuals in our model become increasingly vigilant to signs of an ongoing outbreak, i.e., when θ^{+}_{a} increases, the first disease outbreaks are flattened and delayed (Fig F(a.1)-F(d.1)), compared with average of multiple outcomes of the corresponding biased random walk foraging model described as in Section 2.4 in the main text. When it comes to spatial distribution of active-virus carriers (Fig F(a.3)-F(a.5), F(b.3)-F(b.5), F(c.3)-F(c.5) and F(d.3)-F(d.5)), it appears that sizes of clusters are similar to those in the corresponding biased random walk foraging model simulations (Fig 4 in the main text), while the number of infected agents within each cluster decreases in Fig F.

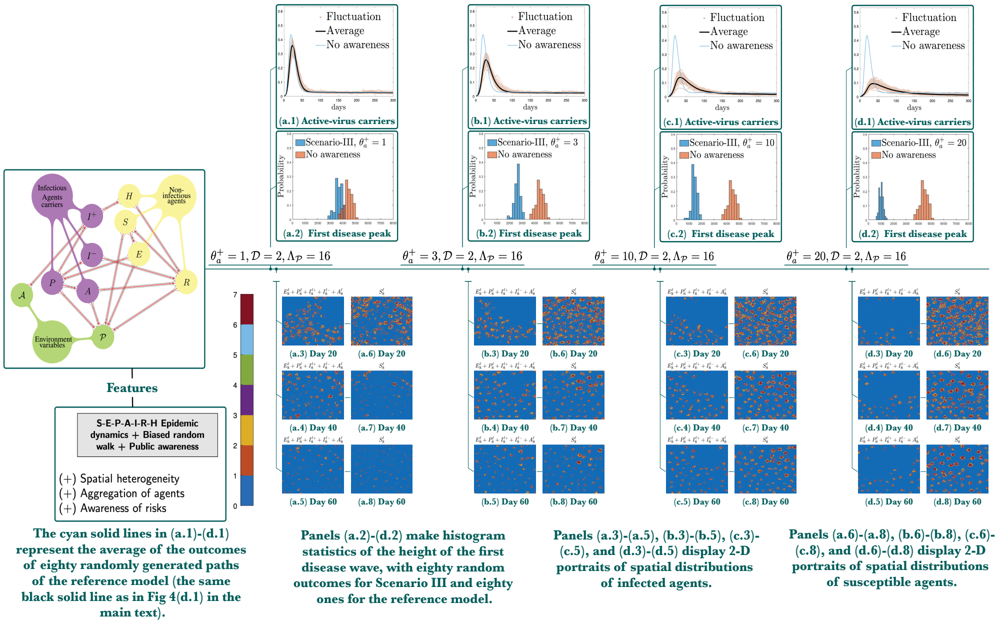

Fig F. Simulations of Scenario III: Reducing infectious contacts. Panels (a.1)-(d.1) compare $\bar{E}^{\ell}(t)+\bar{P}^{\ell}(t)+\bar{A}^{\ell}(t)+\bar{I}^{(-),\ell}(t)+\bar{I}^{(+),\ell}(t)$, where the reference is the biased random walk foraging model without awareness in Section 2.4 in the main text. When the increment of public awareness θ^{+}_{a} increases from 1 to 3 to 10 to 20, individuals become increasingly vigilant to signs of an ongoing epidemic outbreak. As a consequence, the first disease outbreaks are delayed and flattened.

In Fig F(a.1)-F(d.1), agent-based simulations exhibit delayed and suppressed disease peaks as θ^{+}_{a} increases. The same type of transition is also displayed in Fig F(a.2)-F(d.2). Indeed, spread of epidemic decelerates as θ^{+}_{a} increases, at each fixed time (day 20, 40, and 60), as displayed in Fig F(a.6)-F(a.8), F(b.6)-F(b.8), F(c.6)-F(c.8), and

F(d.6)-F(d.8). In particular, at day 20, viewing Fig F(a.6)-F(d.6), area of cavity, i.e. lower density regions of susceptible agents, decreases as θ_a^+ increases; the same type of transitions is displayed in panel sets (a.7)-(d.7) at day 40; and (a.8)-(d.8) at day 60.
