# Supplementary material for: Interacting particle models on the impact of spatially heterogeneous human behavioral factors on dynamics of infectious diseases
Source: PLoS Comput Biol. 2024 Aug 8;20(8):e1012345. doi: 10.1371/journal.pcbi.1012345 (PMC11335169; doi:10.1371/journal.pcbi.1012345)
Supplement: S1 Data — (ZIP) [file pcbi.1012345.s002.zip › Data_Epidemic_Particle_part_1/Description of Data_of_Epidemic_Particle.docx]

**Description of ‘Data_of_Epidemic_Particle’**

1. Code: MATLAB Code package for simulating particle system

**In the main text**

1. Nofield: Simulation results without popularity or awarenss
2. Aggregation: Simulation results with popularity
3. Aware_biased: Simualtion results for Scenrio-I
4. Aware_move: Simualtion results for Scenrio-II-i
5. Aware_uniform: Simualtion results for Scenrio-II-ii

**In Supplementary materials**

1. Aware_P2Ps: Simualtion results for Scenrio-III
2. Nofield_lossless: Simulation results without popularity or awarenss, without waning of immunity
3. Aggregation_scaling: Sensitivity analysis under temporal scaling
