# Supplementary figures and images for: Interacting particle models on the impact of spatially heterogeneous human behavioral factors on dynamics of infectious diseases
Source: PLoS Comput Biol. 2024 Aug 8;20(8):e1012345. doi: 10.1371/journal.pcbi.1012345 (PMC11335169; doi:10.1371/journal.pcbi.1012345)

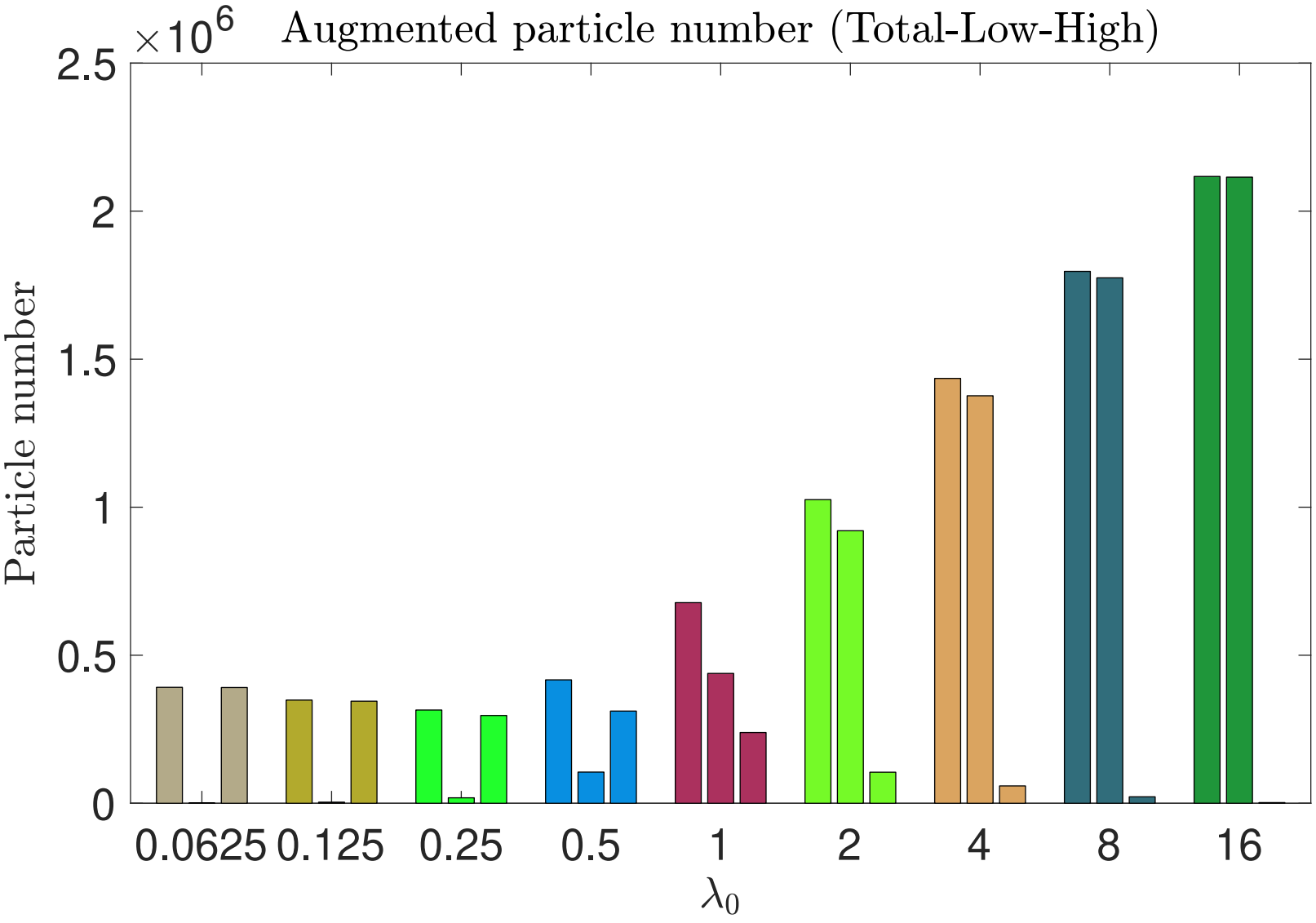

Supplement: S2 Data — (ZIP) [file pcbi.1012345.s003.zip › Data_Epidemic_Particle_part_2/export_fig/additive_particle.pdf]

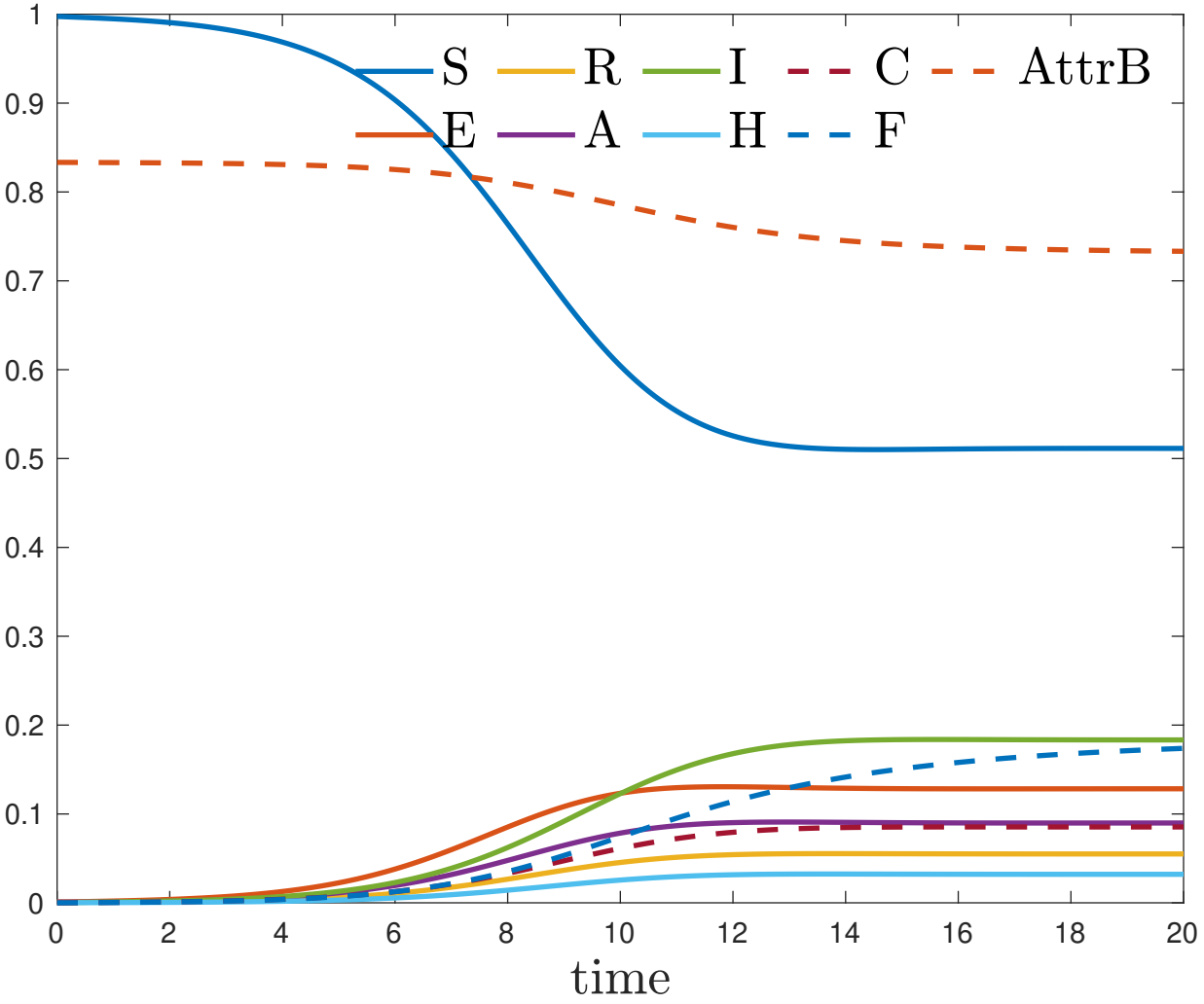

Supplement: S2 Data — (ZIP) [file pcbi.1012345.s003.zip › Data_Epidemic_Particle_part_2/export_fig/epidemic_ETD2CF3.pdf]

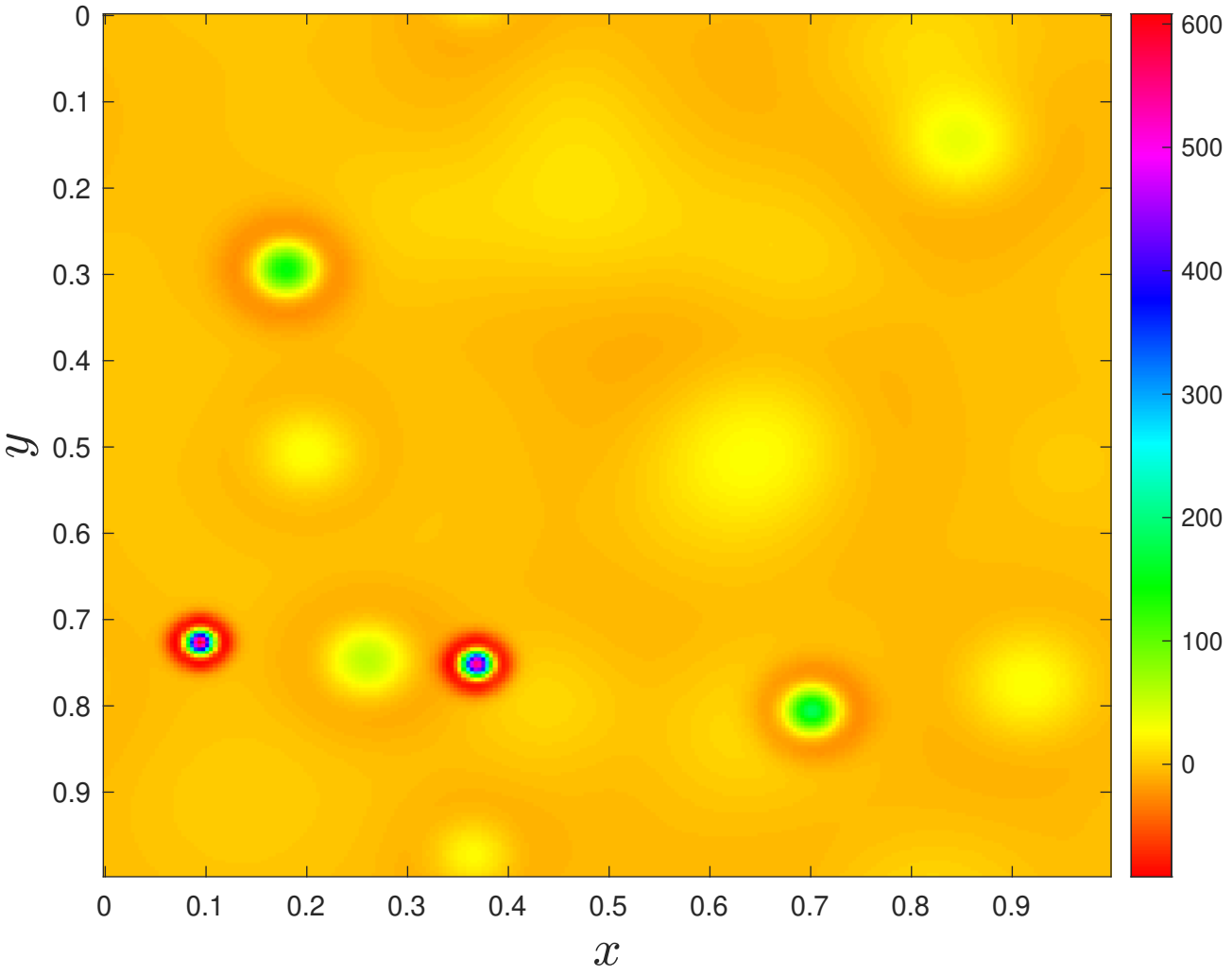

Supplement: S2 Data — (ZIP) [file pcbi.1012345.s003.zip › Data_Epidemic_Particle_part_2/export_fig/FD_256.pdf]

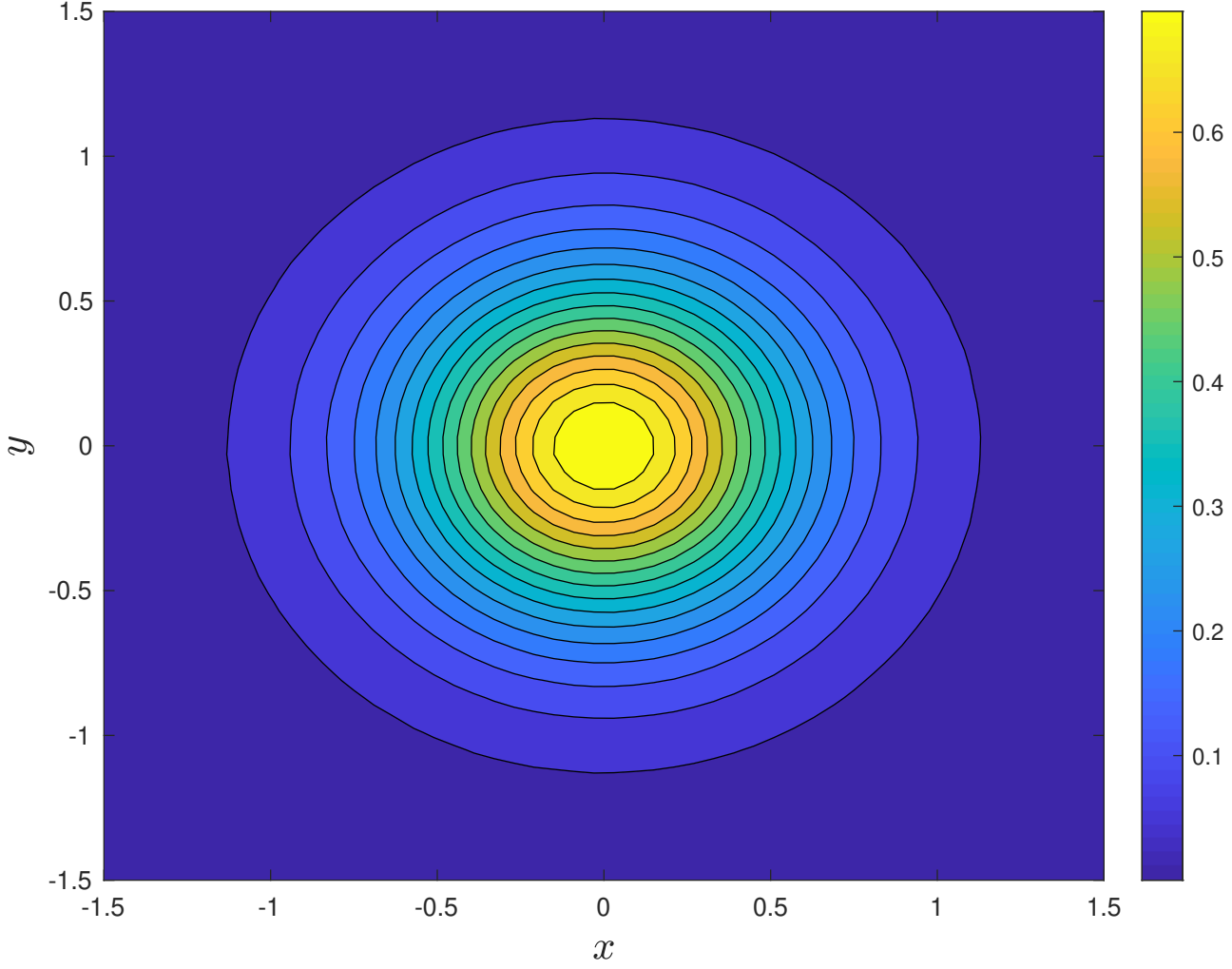

Supplement: S2 Data — (ZIP) [file pcbi.1012345.s003.zip › Data_Epidemic_Particle_part_2/export_fig/orbit/1s_momentum.pdf]

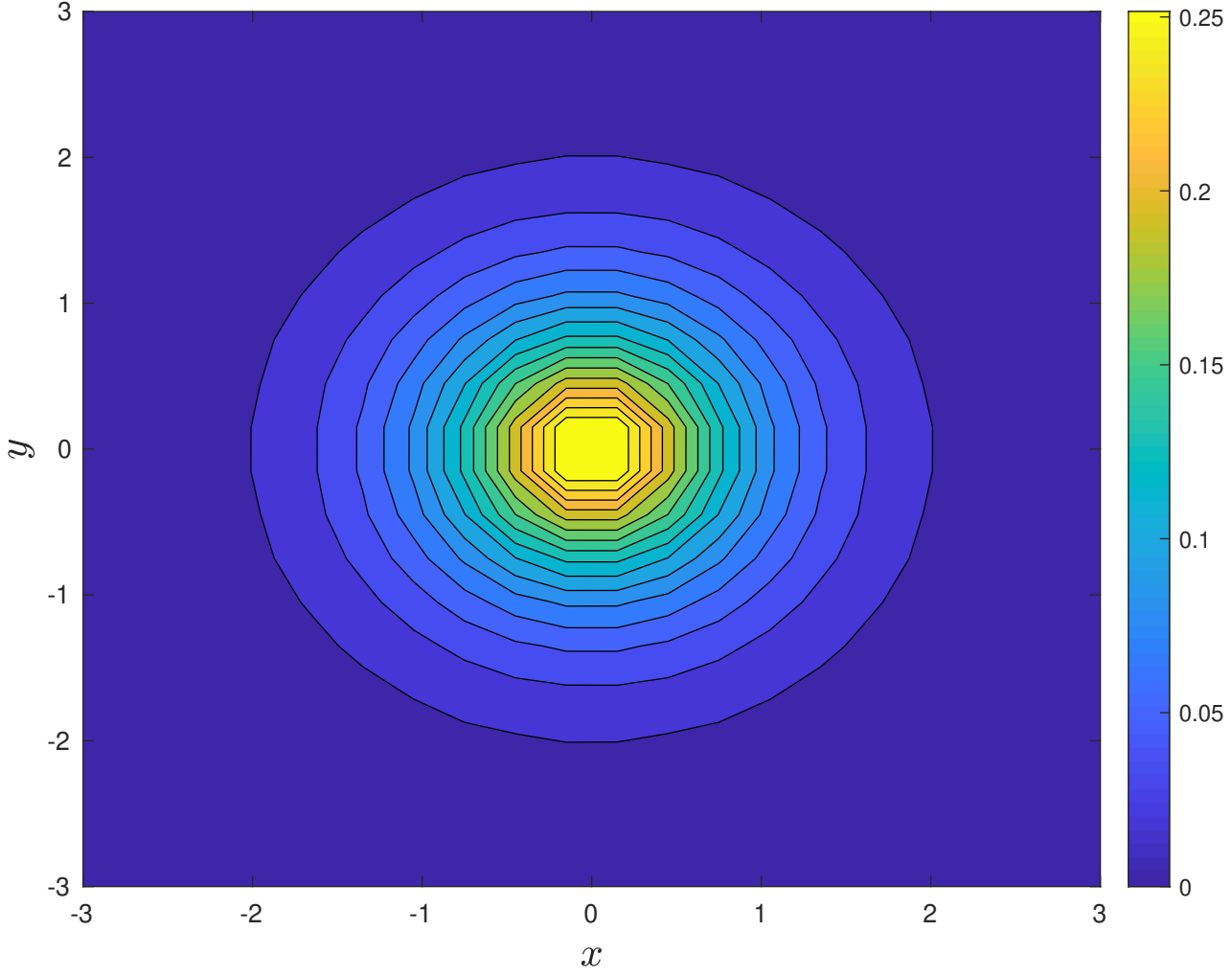

Supplement: S2 Data — (ZIP) [file pcbi.1012345.s003.zip › Data_Epidemic_Particle_part_2/export_fig/orbit/1s_spatial.pdf]

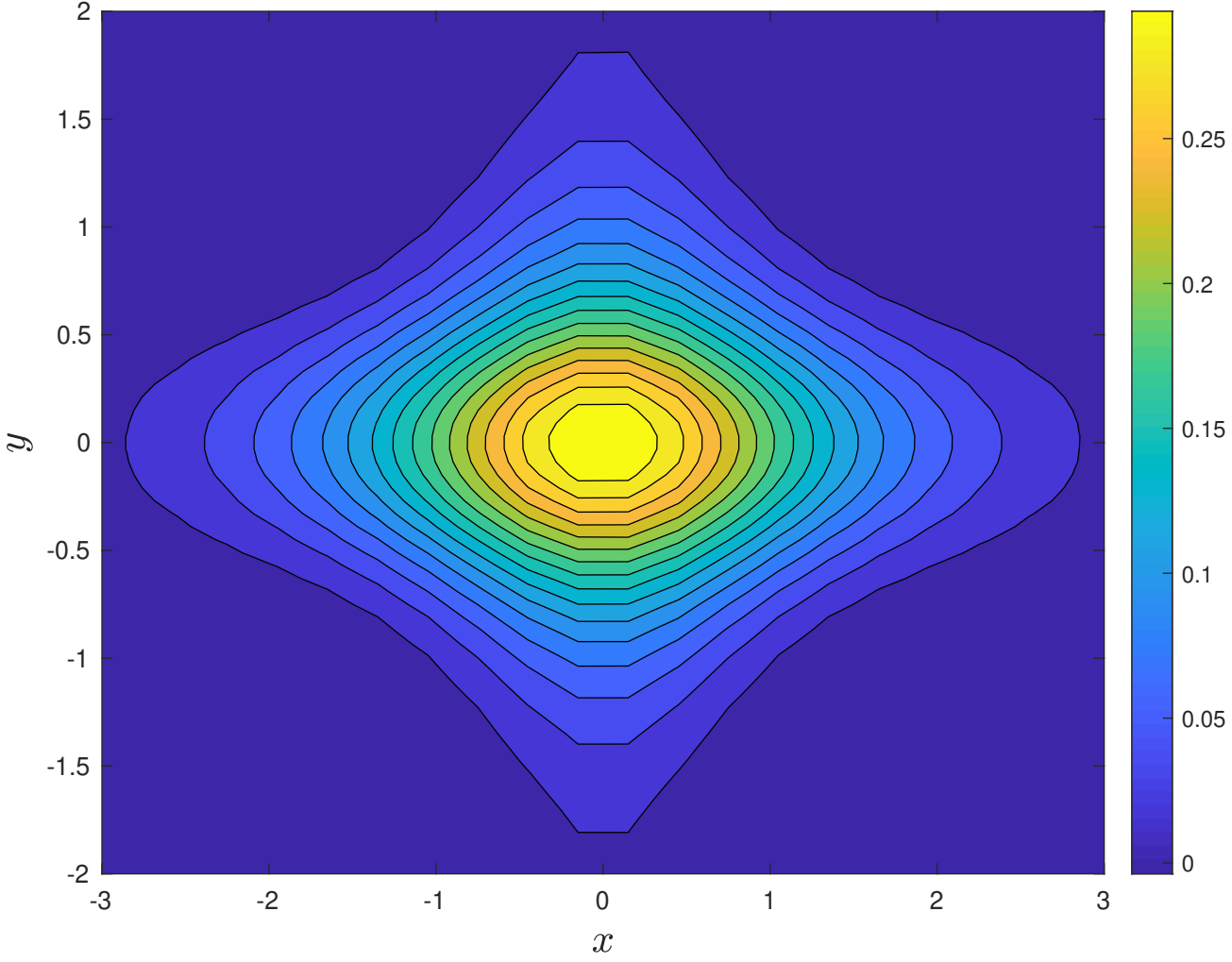

Supplement: S2 Data — (ZIP) [file pcbi.1012345.s003.zip › Data_Epidemic_Particle_part_2/export_fig/orbit/1s_Wigner.pdf]

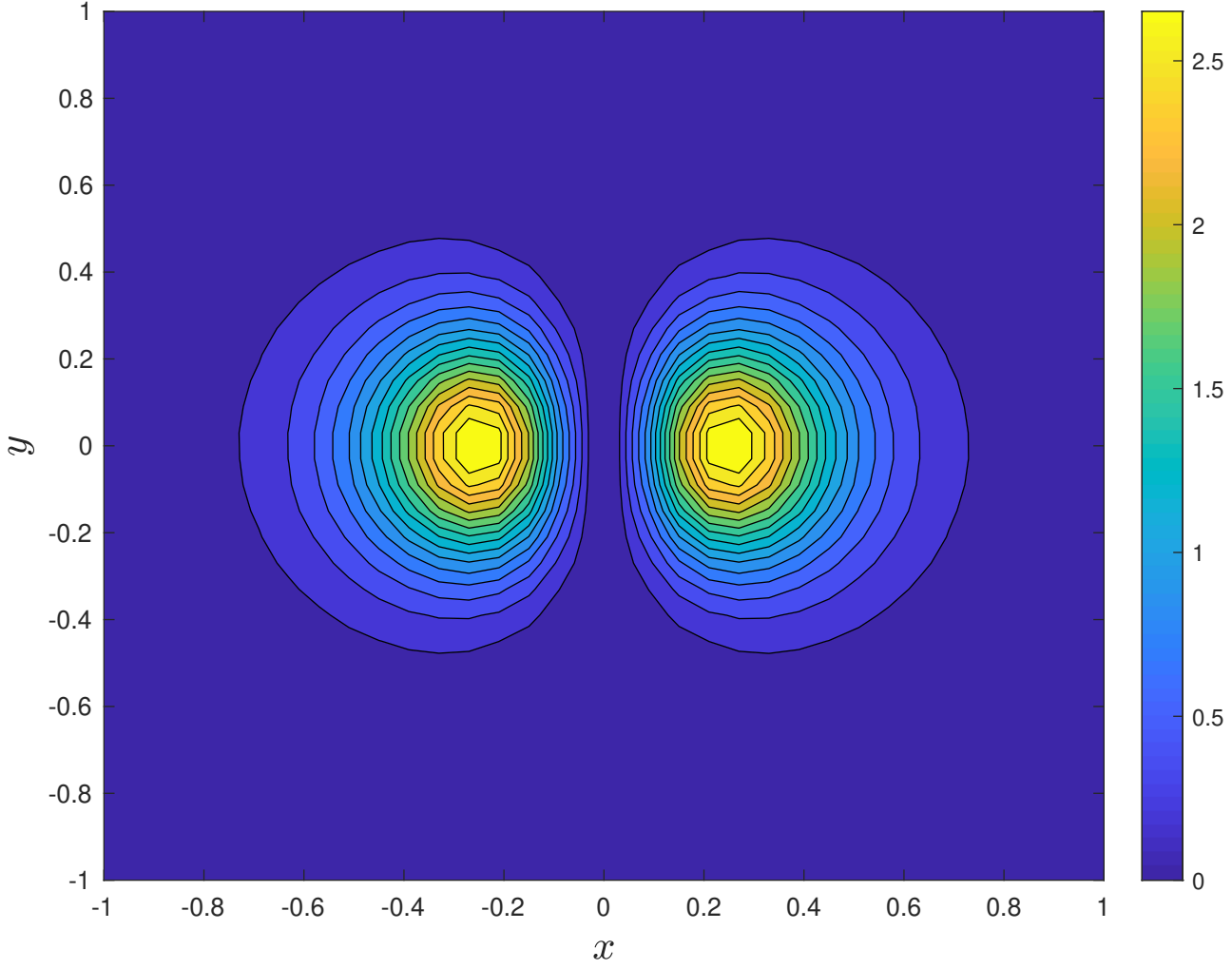

Supplement: S2 Data — (ZIP) [file pcbi.1012345.s003.zip › Data_Epidemic_Particle_part_2/export_fig/orbit/2p_momentum.pdf]

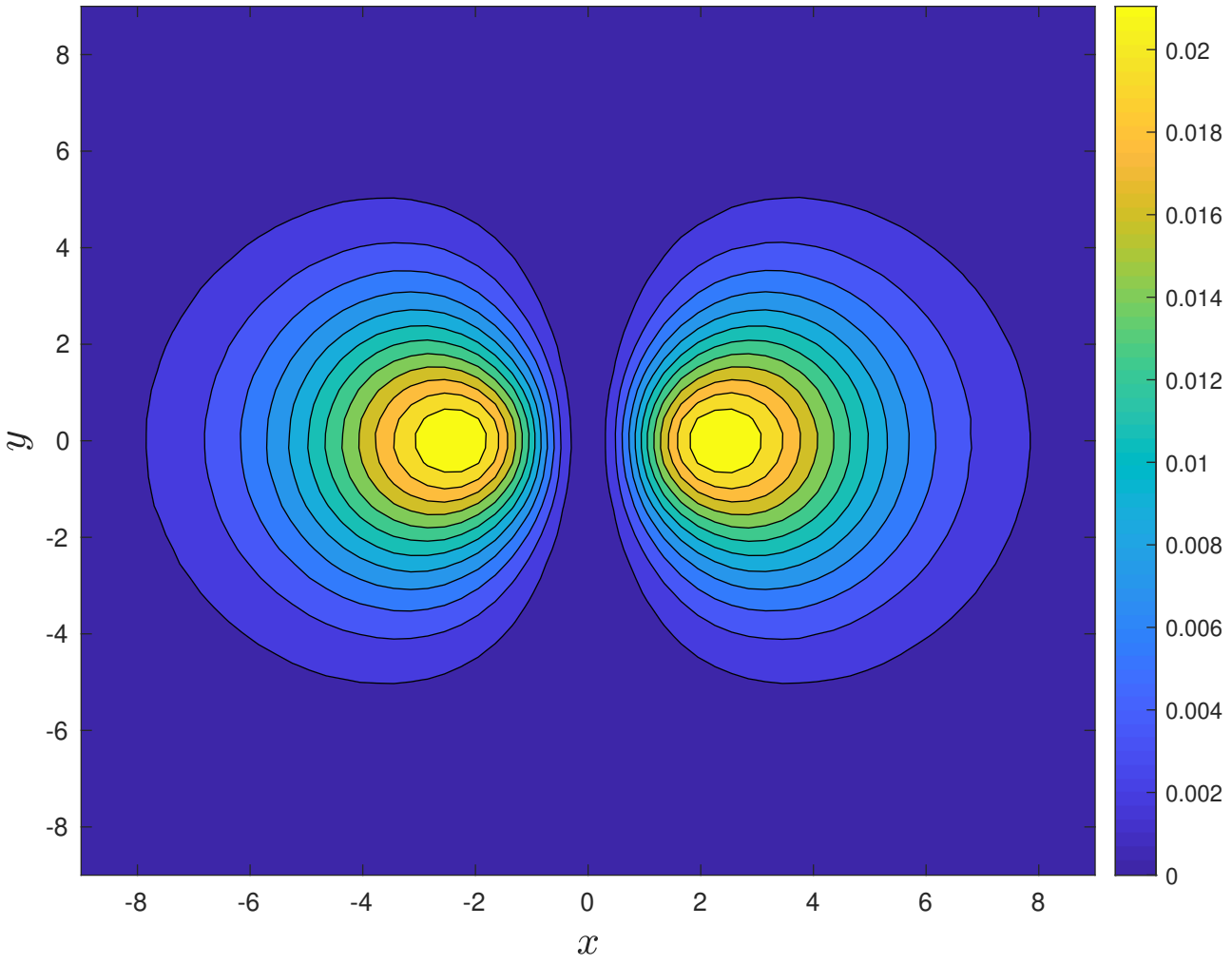

Supplement: S2 Data — (ZIP) [file pcbi.1012345.s003.zip › Data_Epidemic_Particle_part_2/export_fig/orbit/2p_spatial.pdf]

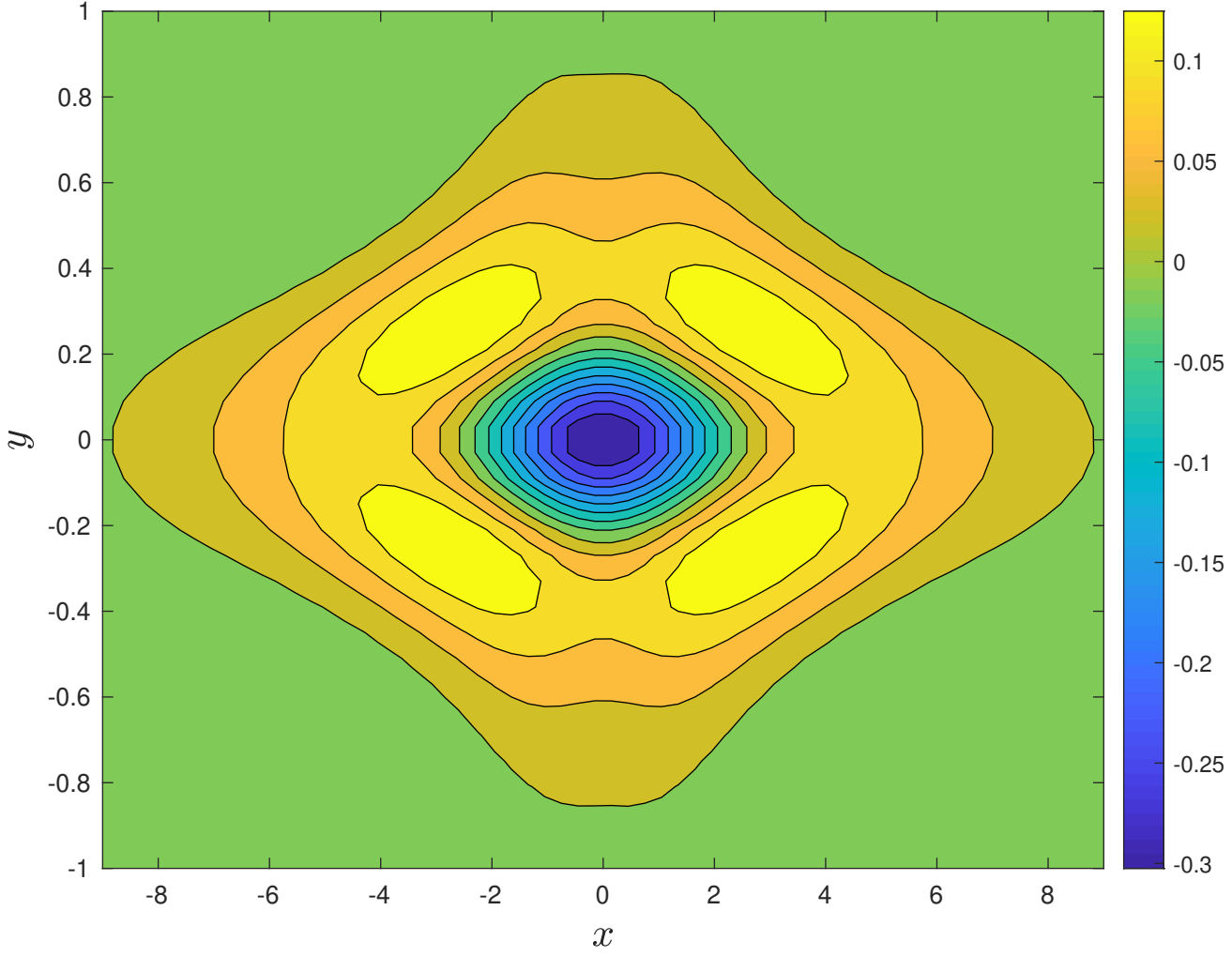

Supplement: S2 Data — (ZIP) [file pcbi.1012345.s003.zip › Data_Epidemic_Particle_part_2/export_fig/orbit/2p_Wigner.pdf]

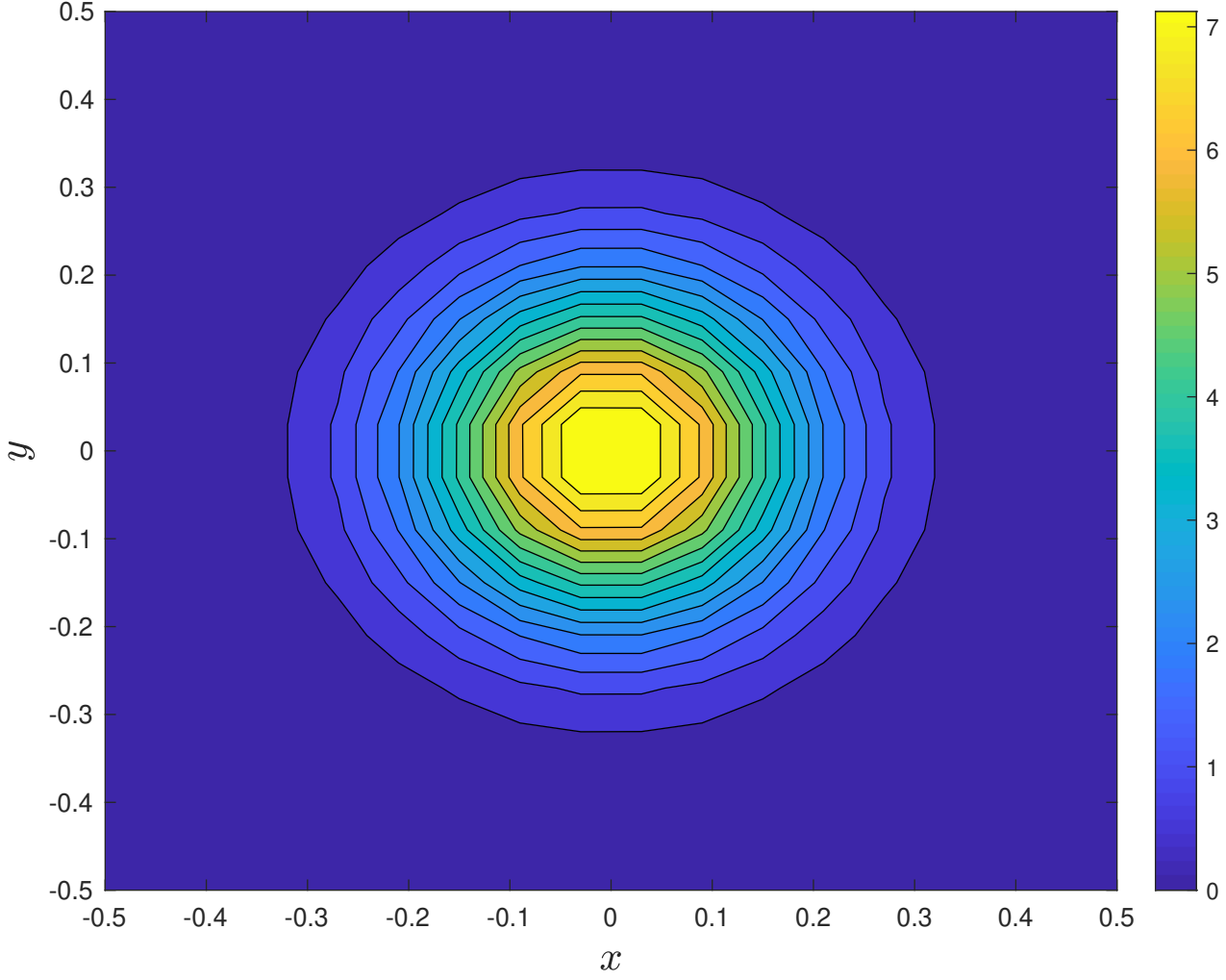

Supplement: S2 Data — (ZIP) [file pcbi.1012345.s003.zip › Data_Epidemic_Particle_part_2/export_fig/orbit/2s_momentum.pdf]

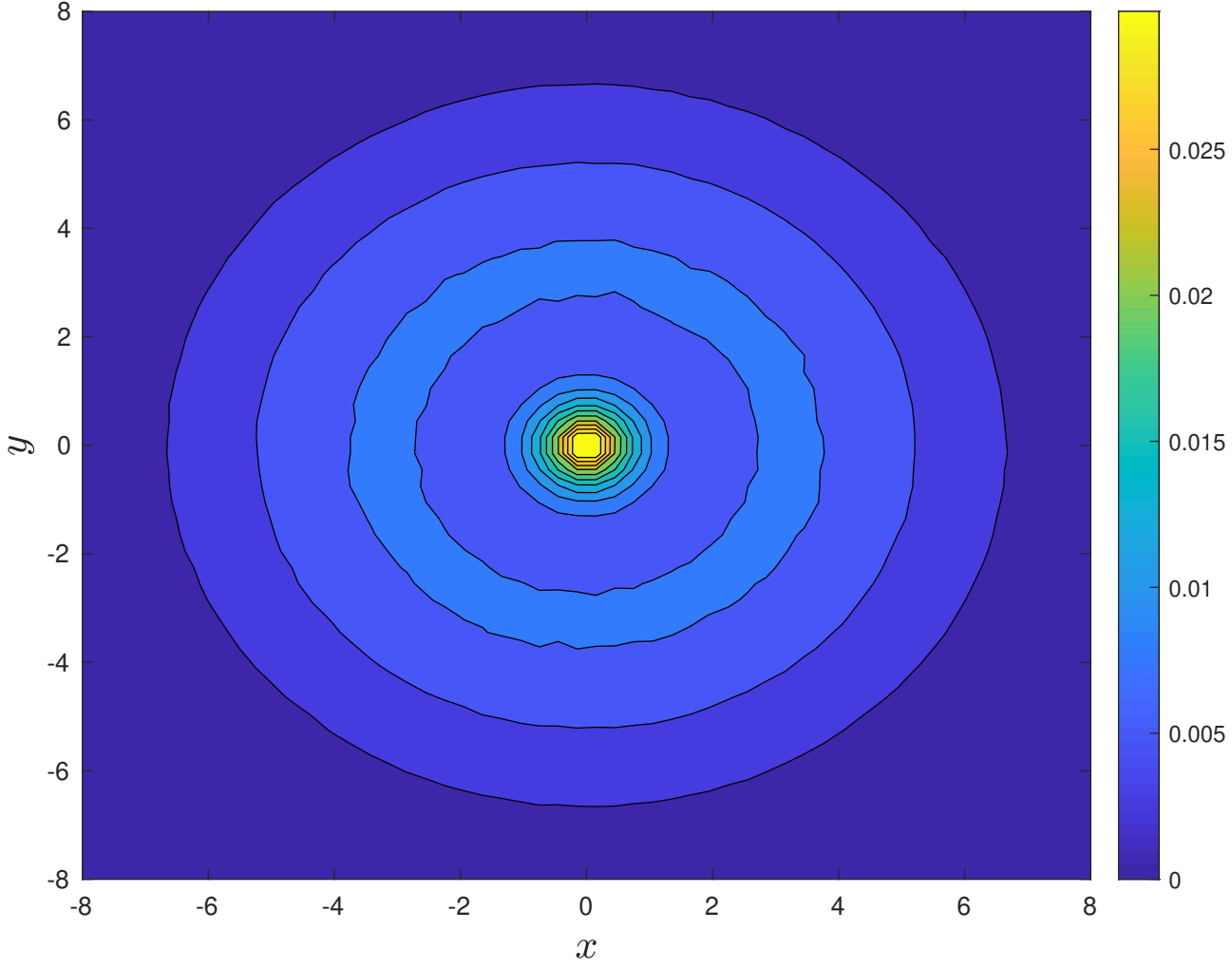

Supplement: S2 Data — (ZIP) [file pcbi.1012345.s003.zip › Data_Epidemic_Particle_part_2/export_fig/orbit/2s_spatial.pdf]

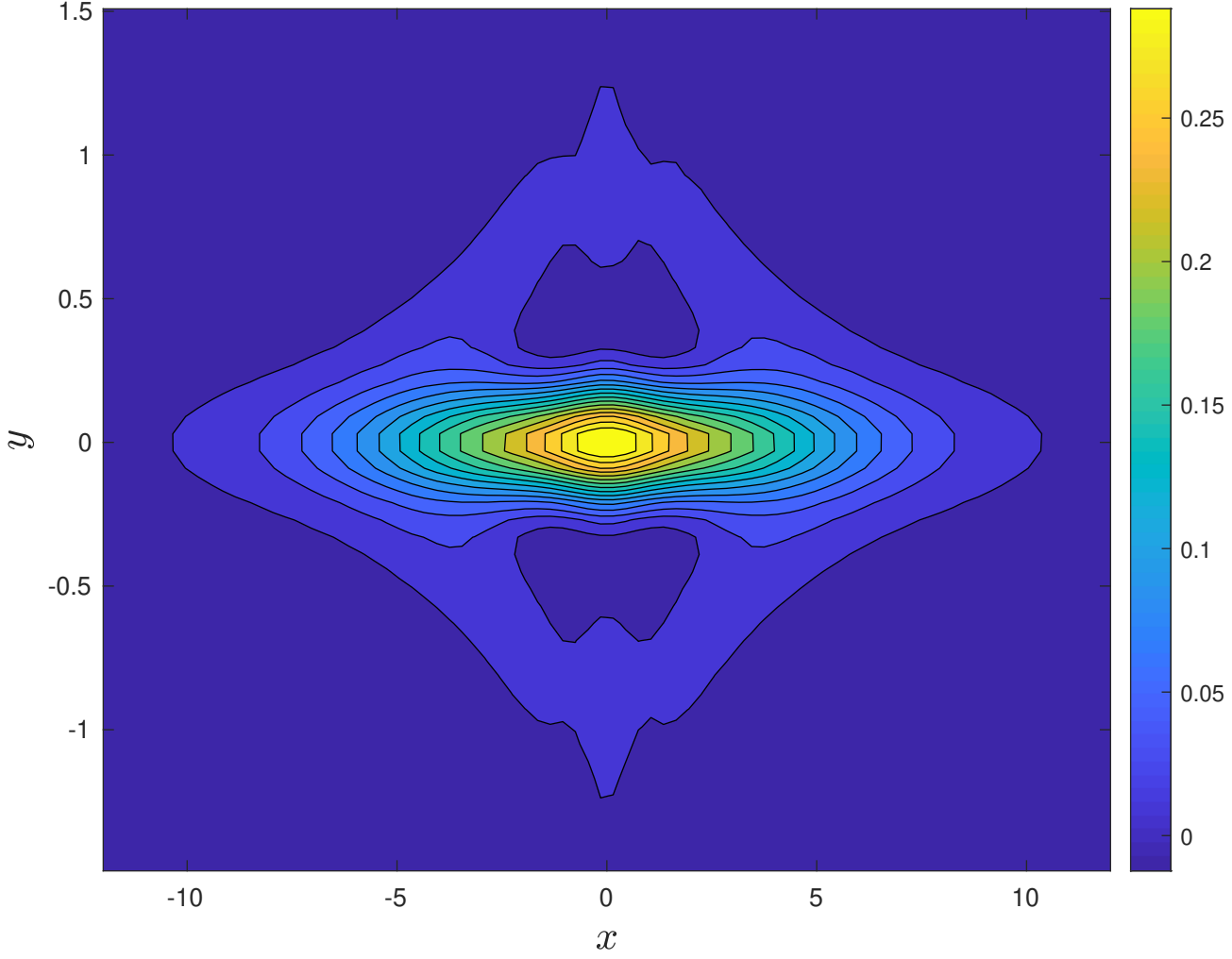

Supplement: S2 Data — (ZIP) [file pcbi.1012345.s003.zip › Data_Epidemic_Particle_part_2/export_fig/orbit/2s_Wigner.pdf]

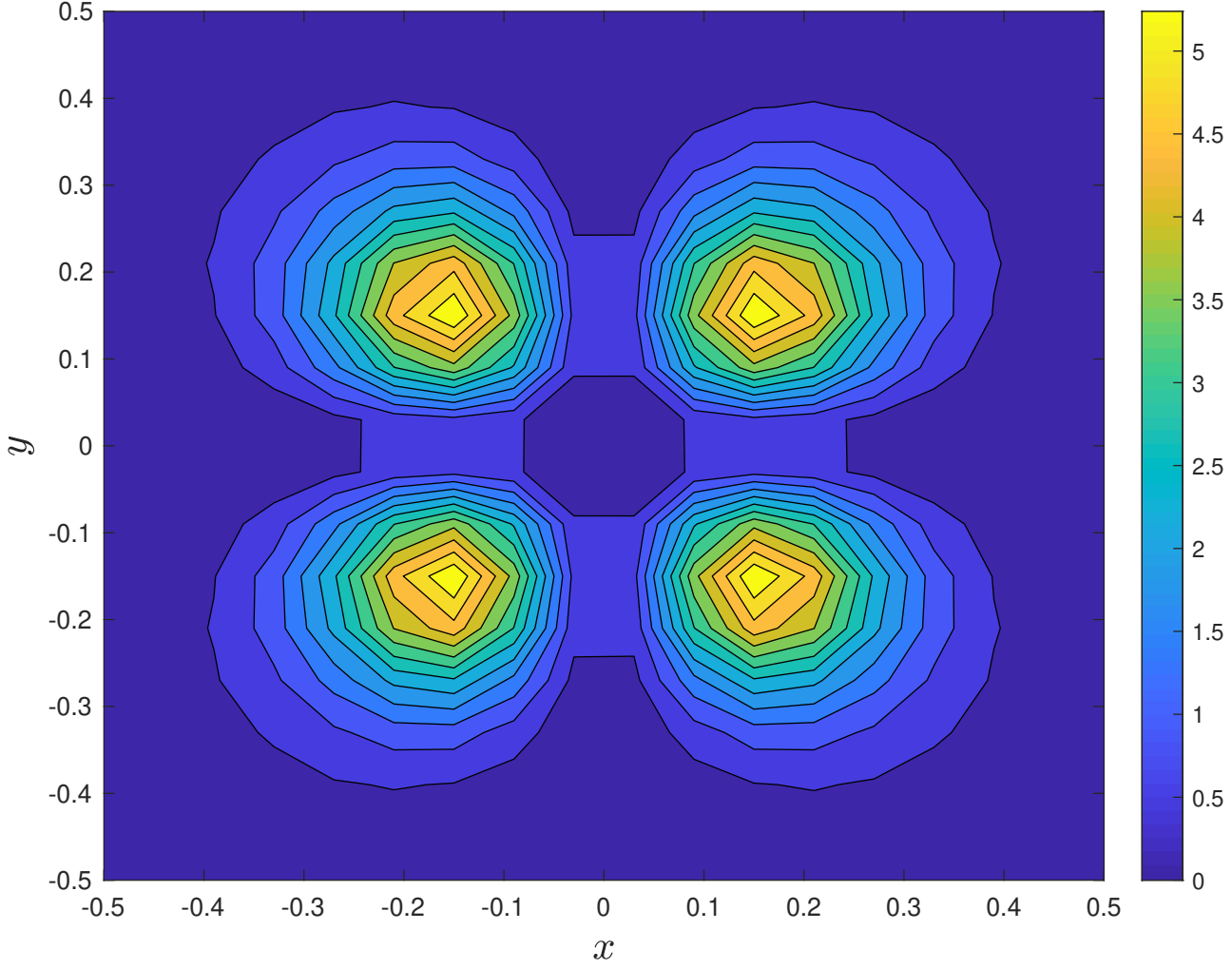

Supplement: S2 Data — (ZIP) [file pcbi.1012345.s003.zip › Data_Epidemic_Particle_part_2/export_fig/orbit/3d_momental.pdf]

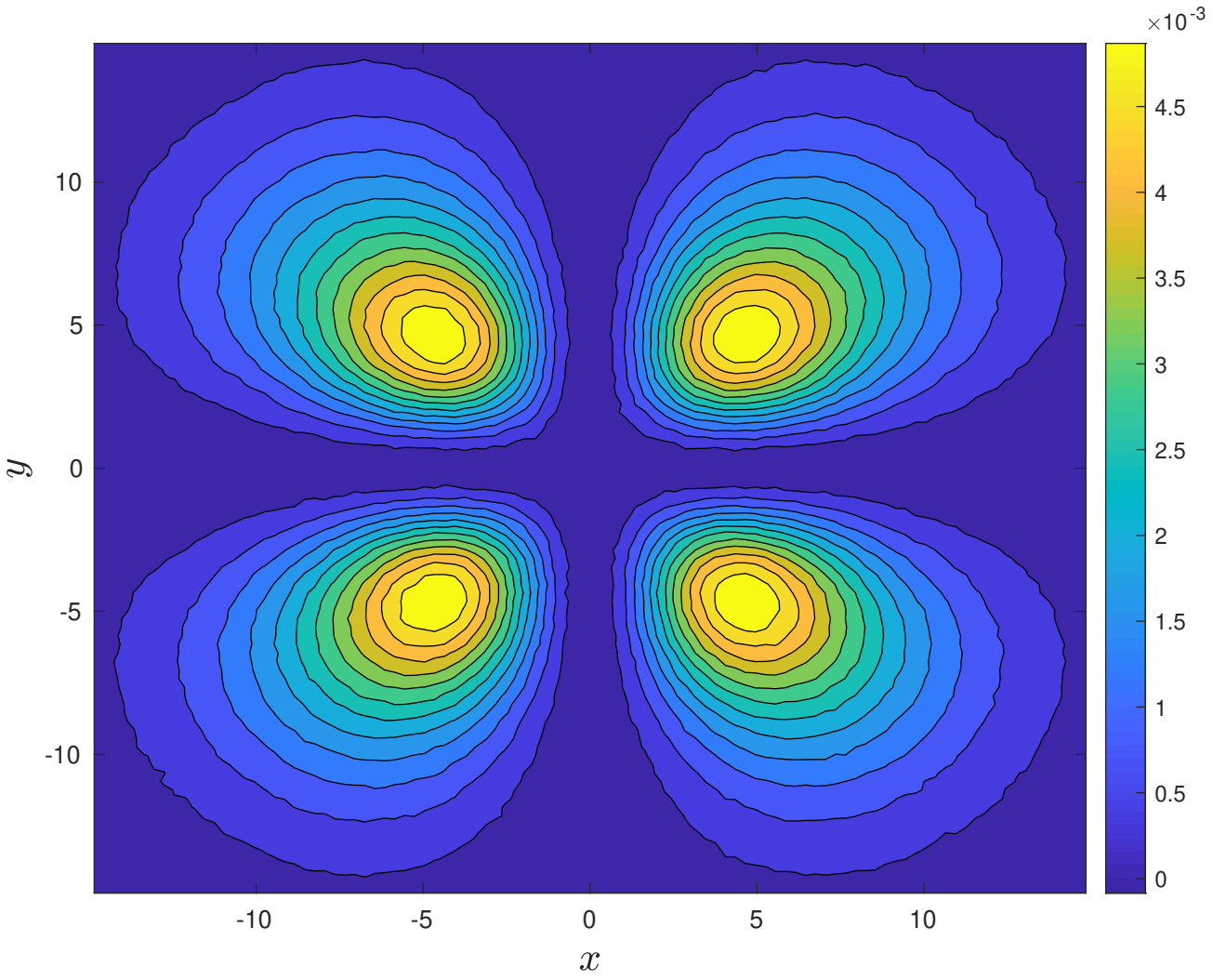

Supplement: S2 Data — (ZIP) [file pcbi.1012345.s003.zip › Data_Epidemic_Particle_part_2/export_fig/orbit/3d_spatial.pdf]

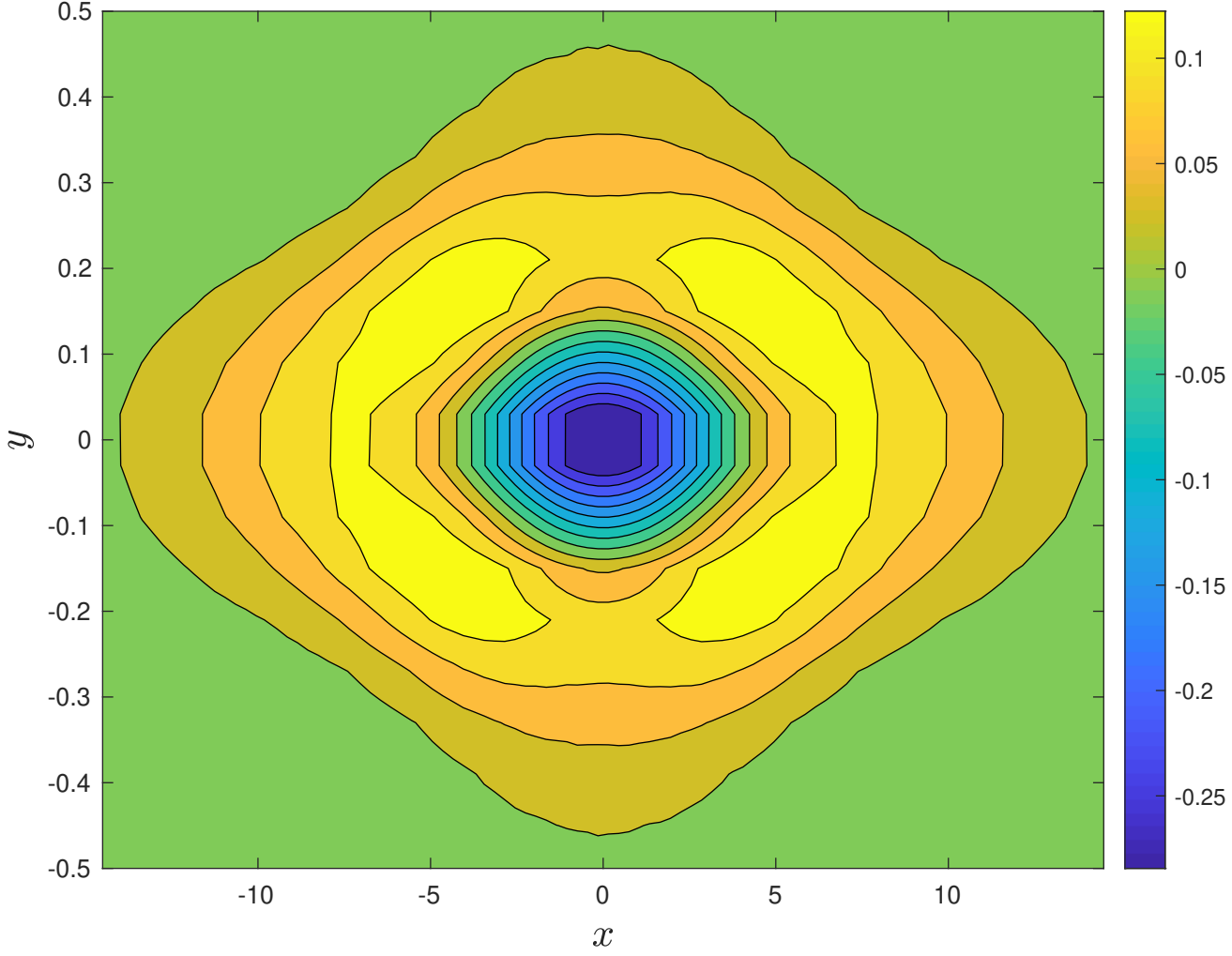

Supplement: S2 Data — (ZIP) [file pcbi.1012345.s003.zip › Data_Epidemic_Particle_part_2/export_fig/orbit/3d_Wigner.pdf]

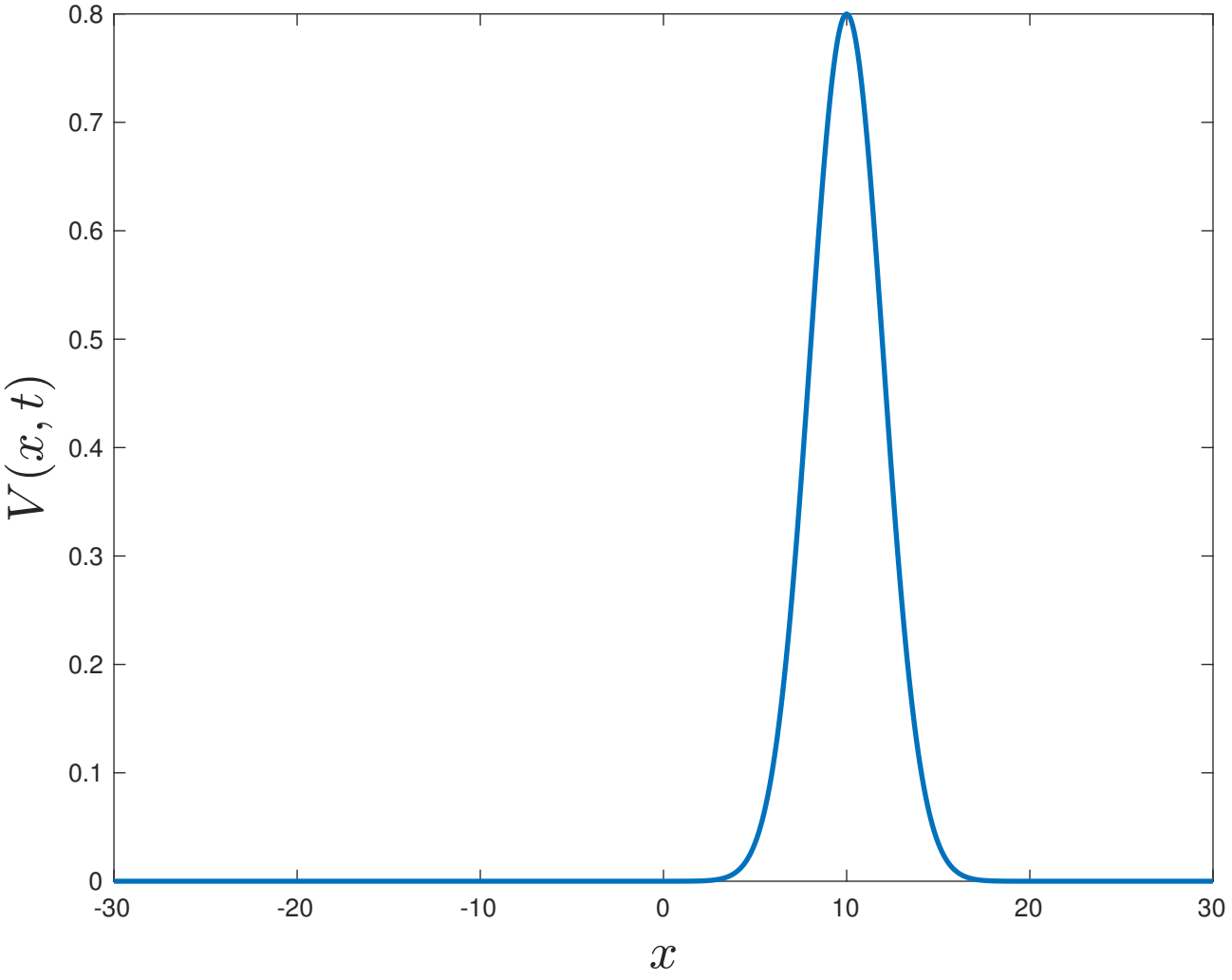

Supplement: S2 Data — (ZIP) [file pcbi.1012345.s003.zip › Data_Epidemic_Particle_part_2/export_fig/potential.pdf]

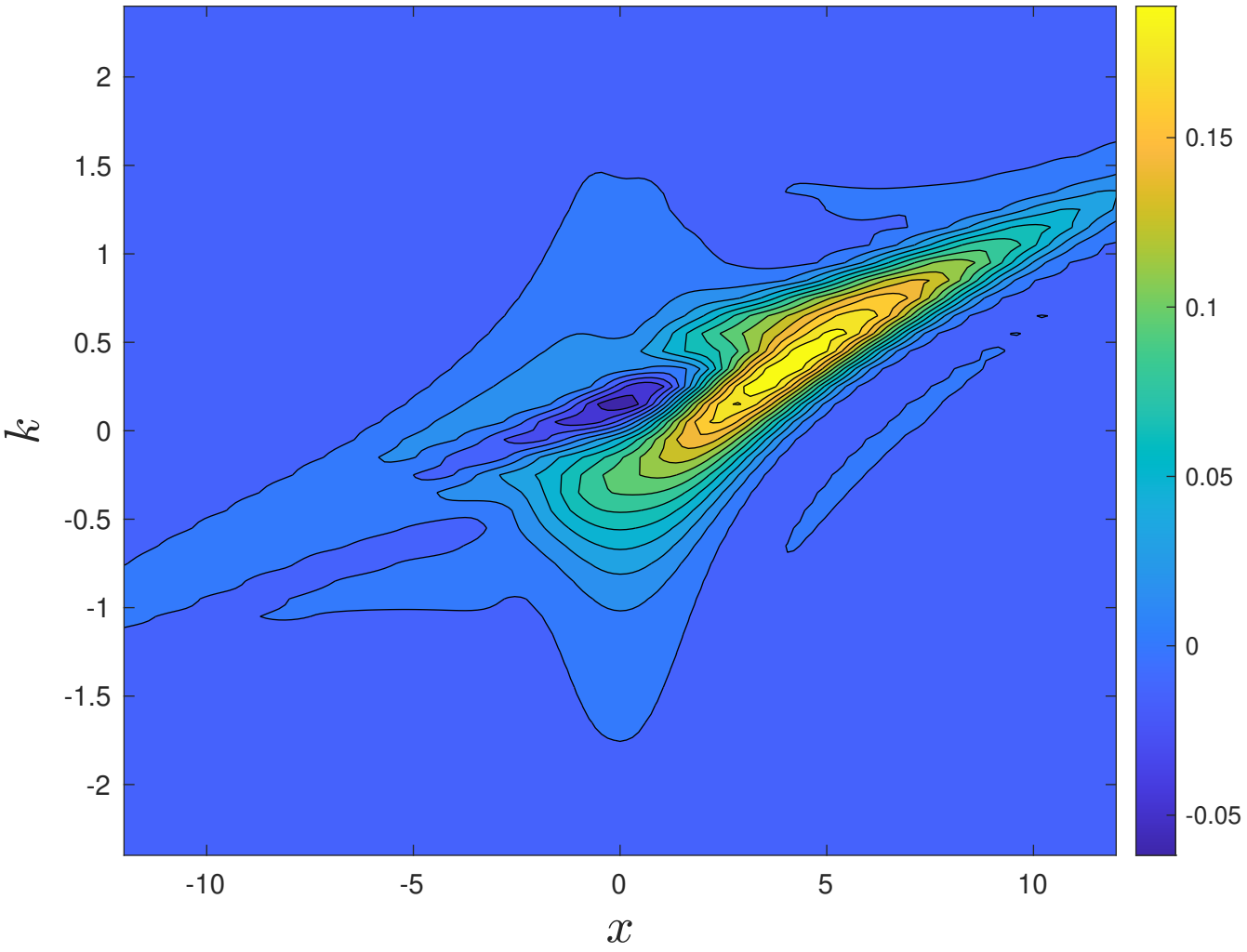

Supplement: S2 Data — (ZIP) [file pcbi.1012345.s003.zip › Data_Epidemic_Particle_part_2/export_fig/redist_asm.pdf]

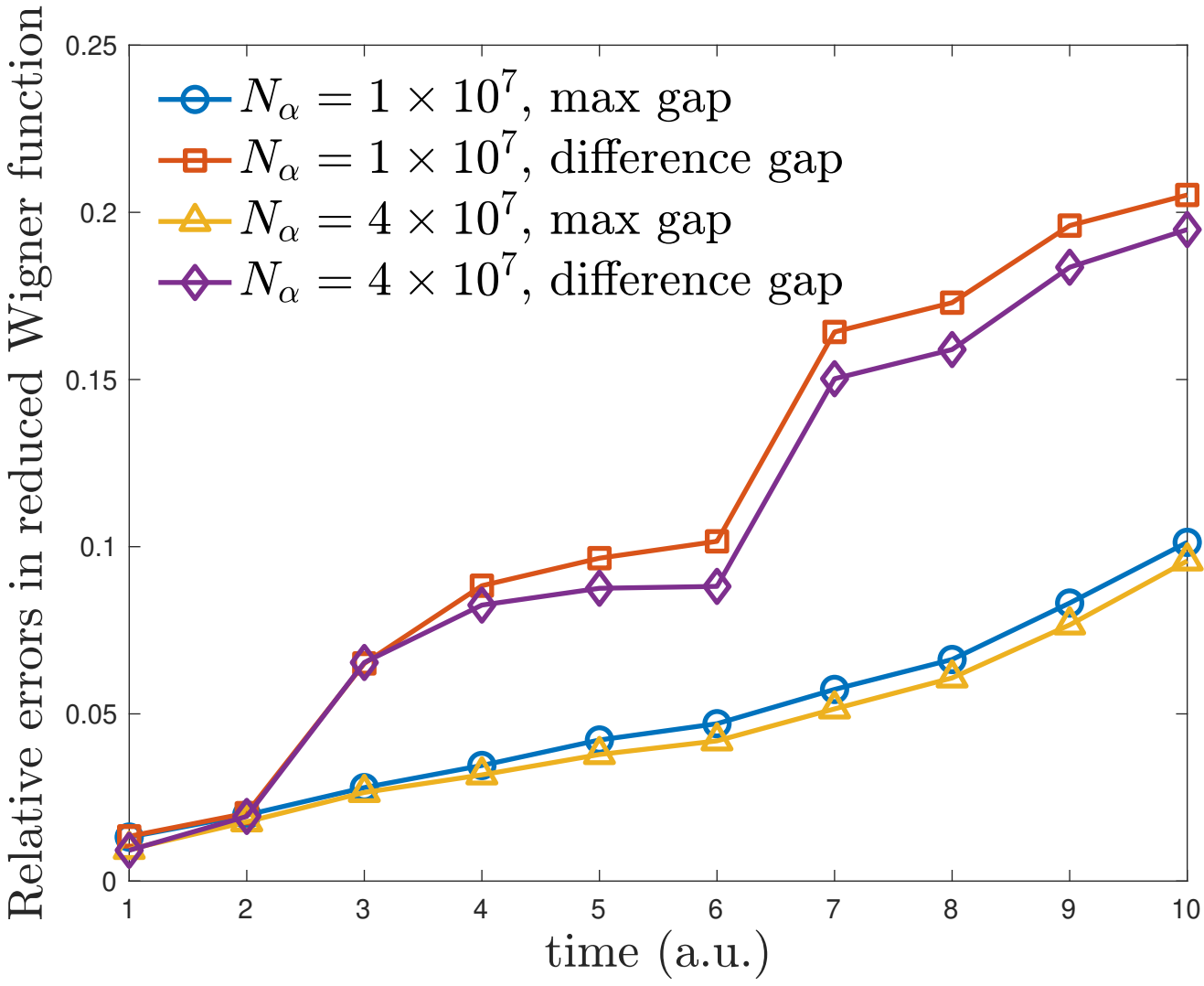

Supplement: S2 Data — (ZIP) [file pcbi.1012345.s003.zip › Data_Epidemic_Particle_part_2/export_fig/redist_err_evo_comp.pdf]

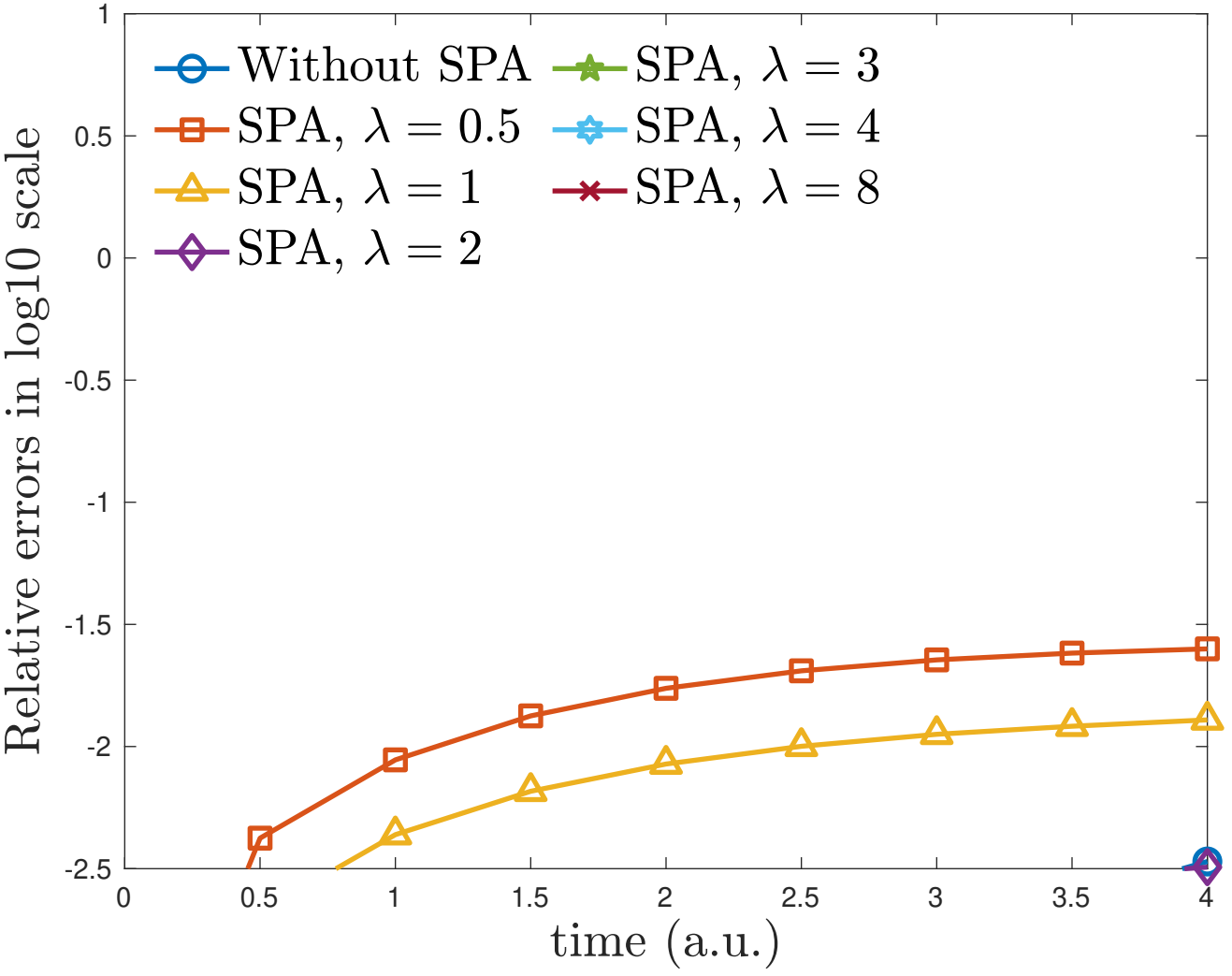

Supplement: S2 Data — (ZIP) [file pcbi.1012345.s003.zip › Data_Epidemic_Particle_part_2/export_fig/redist_err_evo_MC.pdf]

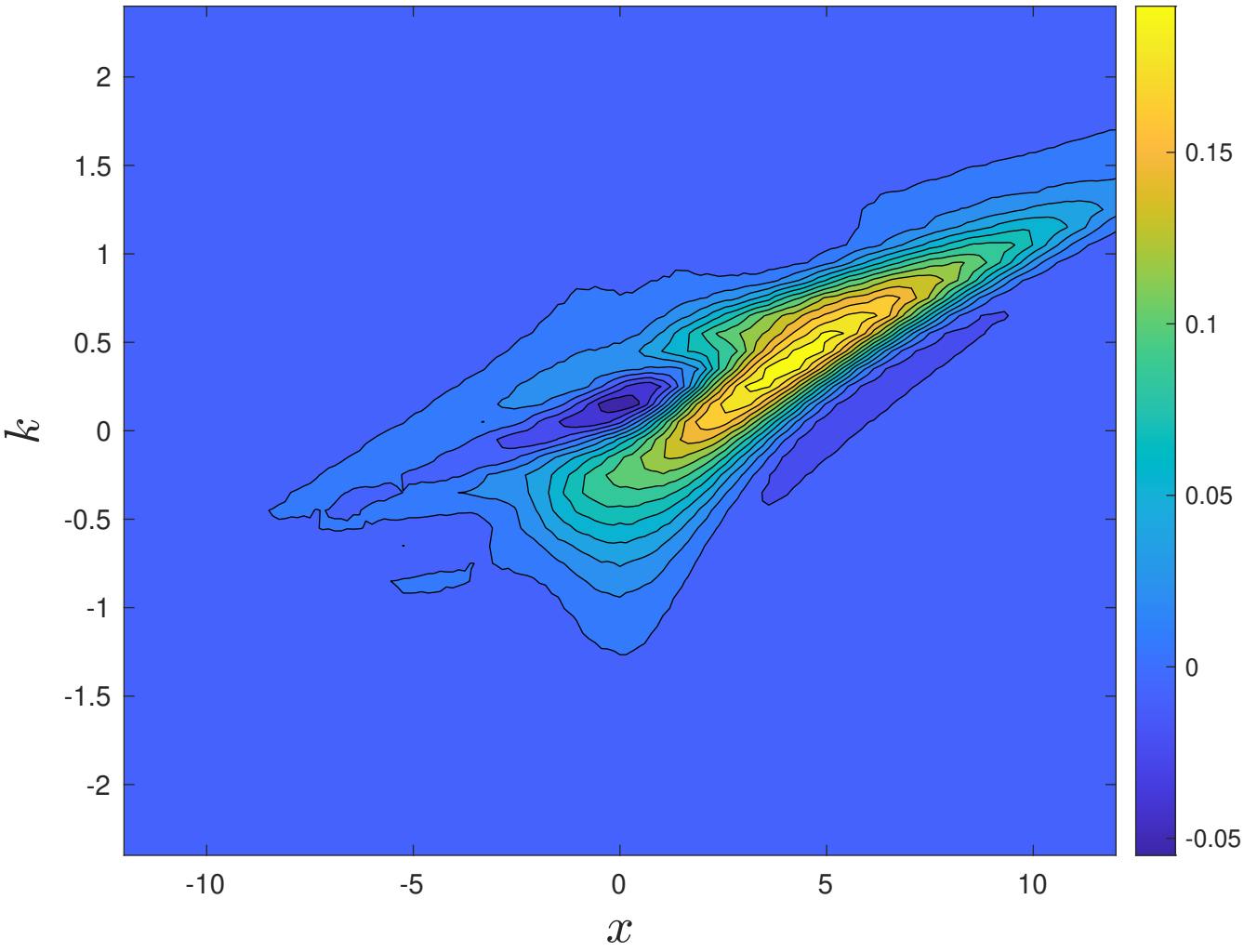

Supplement: S2 Data — (ZIP) [file pcbi.1012345.s003.zip › Data_Epidemic_Particle_part_2/export_fig/redist_spade.pdf]

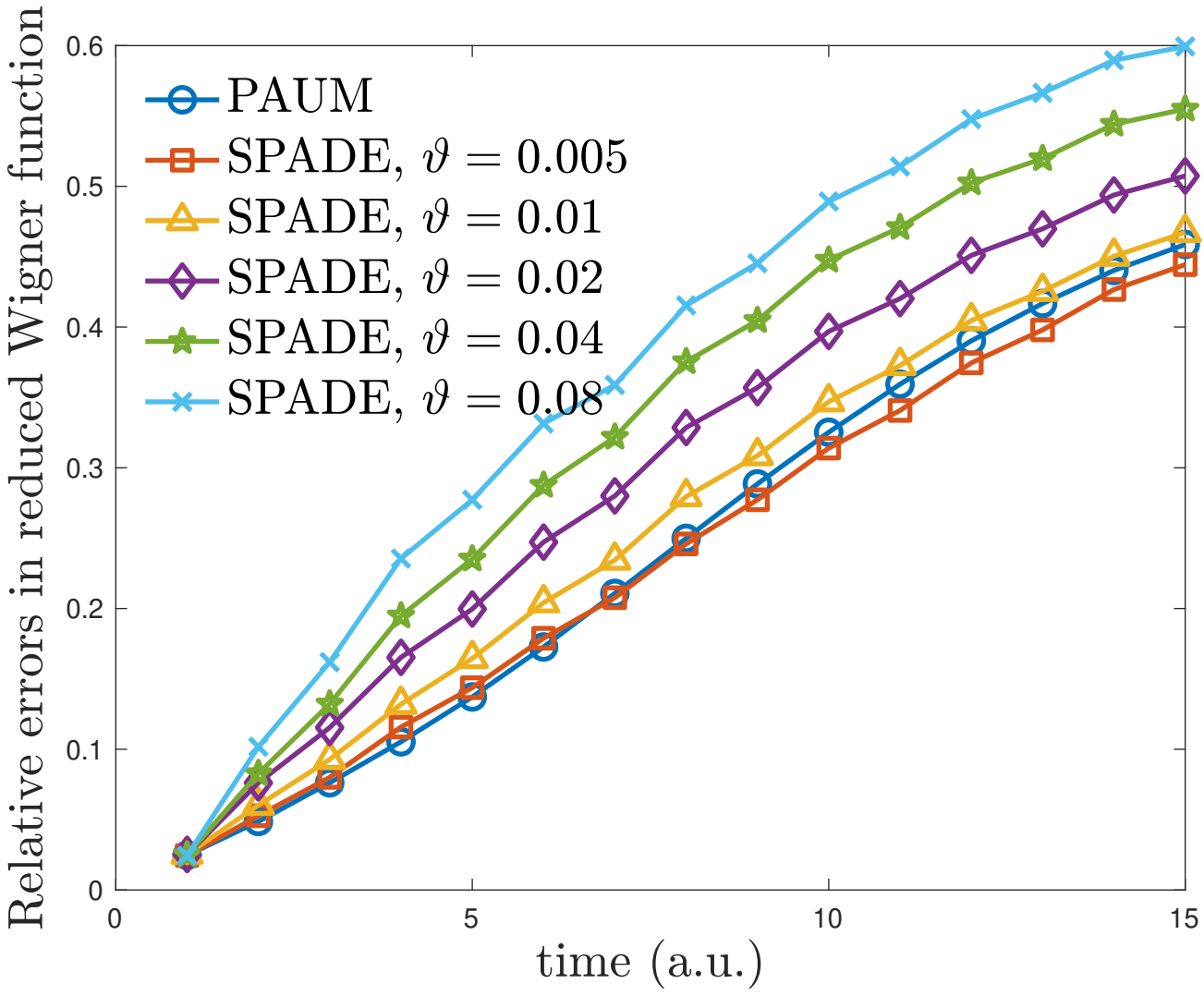

Supplement: S2 Data — (ZIP) [file pcbi.1012345.s003.zip › Data_Epidemic_Particle_part_2/export_fig/redist_x_err_evo_theta.pdf]

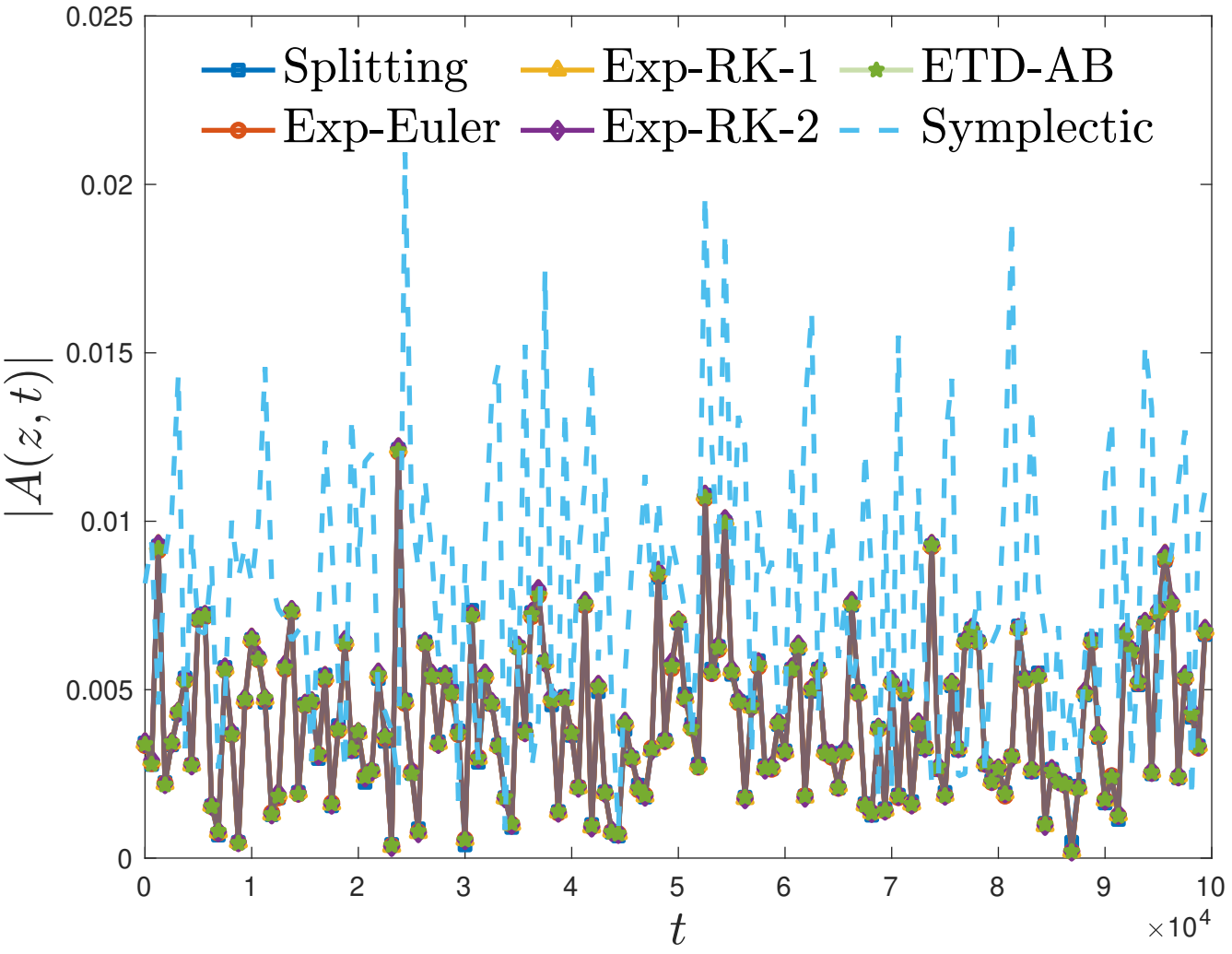

Supplement: S2 Data — (ZIP) [file pcbi.1012345.s003.zip › Data_Epidemic_Particle_part_2/export_fig/wm_norm_diff.pdf]

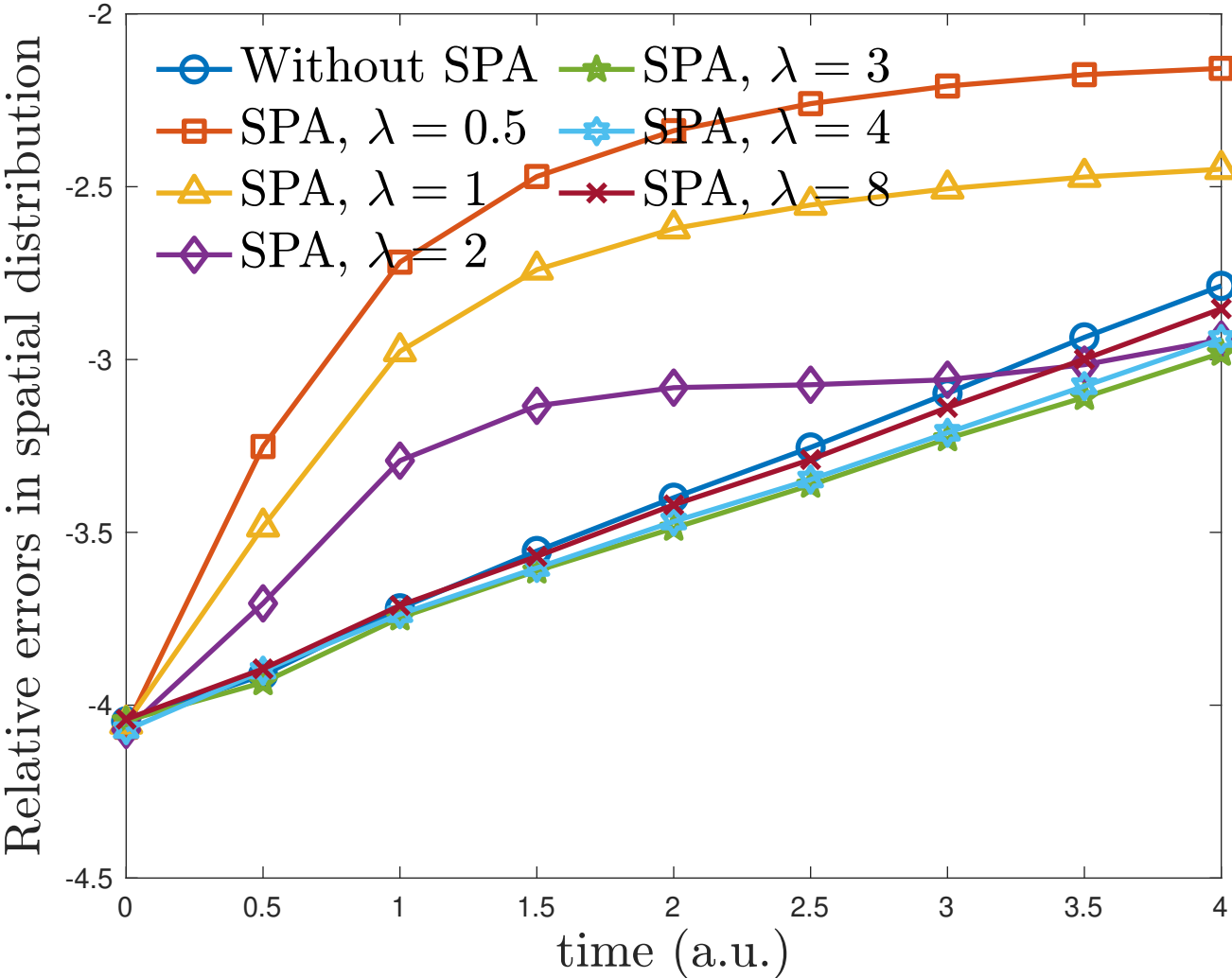

Supplement: S2 Data — (ZIP) [file pcbi.1012345.s003.zip › Data_Epidemic_Particle_part_2/export_fig/xdist_err_evo_MC.pdf]
